# A genome-wide transcriptome map of pistachio (*Pistacia vera* L.) provides novel insights into salinity-related genes and marker discovery

**DOI:** 10.1186/s12864-017-3989-7

**Published:** 2017-08-17

**Authors:** Maryam Moazzzam Jazi, Seyed Mahdi Seyedi, Esmaeil Ebrahimie, Mansour Ebrahimi, Gianluca De Moro, Christopher Botanga

**Affiliations:** 1 Plant Biotechnology Department, National Institute of Genetic Engineering and Biotechnology, Tehran, Iran; 2 School of Medicine, The University of Adelaide, Adelaide, Australia; 3 Institute of Biotechnology, Shiraz University, Shiraz, Iran; 4 Division of Information Technology, Engineering and the Environment, School of Information Technology and Mathematical Sciences, University of South Australia, Adelaide, Australia; 5 School of Biological Sciences, Faculty of Science and Engineering, Flinders University, Adelaide, Australia; 6 Department of Biology, University of Qom, Qom, Iran; 7 Center of Marine Sciences (CCMAR), University of Algarve, Faro, Portugal; 8 Department of Biological Sciences, Chicago State University, Chicago, IL USA

## Abstract

**Background:**

Pistachio (*Pistacia vera* L.) is one of the most important commercial nut crops worldwide. It is a salt-tolerant and long-lived tree, with the largest cultivation area in Iran. Climate change and subsequent increased soil salt content have adversely affected the pistachio yield in recent years. However, the lack of genomic/global transcriptomic sequences on *P. vera* impedes comprehensive researches at the molecular level. Hence, whole transcriptome sequencing is required to gain insight into functional genes and pathways in response to salt stress.

**Results:**

RNA sequencing of a pooled sample representing 24 different tissues of two pistachio cultivars with contrasting salinity tolerance under control and salt treatment by Illumina Hiseq 2000 platform resulted in 368,953,262 clean 100 bp paired-ends reads (90 Gb). Following creating several assemblies and assessing their quality from multiple perspectives, we found that using the annotation-based metrics together with the length-based parameters allows an improved assessment of the transcriptome assembly quality, compared to the solely use of the length-based parameters. The generated assembly by Trinity was adopted for functional annotation and subsequent analyses. In total, 29,119 contigs annotated against all of five public databases, including NR, UniProt, TAIR10, KOG and InterProScan. Among 279 KEGG pathways supported by our assembly, we further examined the pathways involved in the plant hormone biosynthesis and signaling as well as those to be contributed to secondary metabolite biosynthesis due to their importance under salinity stress. In total, 11,337 SSRs were also identified, which the most abundant being dinucleotide repeats. Besides, 13,097 transcripts as candidate stress-responsive genes were identified. Expression of some of these genes experimentally validated through quantitative real-time PCR (qRT-PCR) that further confirmed the accuracy of the assembly. From this analysis, the contrasting expression pattern of *NCED3* and *SOS1* genes were observed between salt-sensitive and salt-tolerant cultivars.

**Conclusion:**

This study, as the first report on the whole transcriptome survey of *P. vera*, provides important resources and paves the way for functional and comparative genomic studies on this major tree to discover the salinity tolerance-related markers and stress response mechanisms for breeding of new pistachio cultivars with more salinity tolerance.

**Electronic supplementary material:**

The online version of this article (doi:10.1186/s12864-017-3989-7) contains supplementary material, which is available to authorized users.

## Background

Pistachio belongs to the Anacardiaceae family and consists of at least 11 species, which *P. vera* is the only cultivated and economically important species [1]; the other species are mostly used as rootstocks for cultivation of *P. vera*. It has a long history of cultivation (3000–4000 years) in Iran [2]. Currently, Iran, United States, Turkey, and Syria are the major pistachio producers in the world; among them Iran is ranked first with an average 298, 838.67 tons of production from 1994 to 2014 [3]. Moreover, different parts of plant, including flower, leaf, seed and resins derived from stem, have pharmacological properties like antimicrobial, antioxidant and anti-inflammatory activities [4, 5].

In addition to its significant economic, nutritional, and medicinal values, *P. vera* is highly adaptable to abiotic stresses and considered as a tolerant species against drought and salt stresses, making it an ideal candidate for reforestation in arid and salinized zones [6, 7]. Although pistachio is categorized as a salt-tolerant glycophyte species, its yield is dramatically constrained under the high salinity conditions [8, 9]. Due to global warming and drought in the last decade, forest area of wild pistachio in Iran has dramatically decreased, which has negative economic, environmental and social impacts. High temperature and low precipitation is responsible for the reduction of soil water storage, and increase in salt content, which is the most important limiting factor for the growth of rainfed trees. Therefore, there is an urgent need for the conservation and sustainable management of this major species to warrant prolonged growth and productivity periods. However, little attention has paid to this nut crop from the cellular molecular point of view that resulting in only 1329 expressed sequence tag (EST) sequences being in the National Center for Biotechnology Information (NCBI) (NCBI, search term “pistachio”, as of December, 2016), and no reference genome. It has been very recently reported the genome survey of *P. vera* cv. Siirt using Illumina sequencing to discover simple sequence repeats (SSR) within the assembled genome, which 59,280 SSR motifs were obtained and 206 SSRs applied to characterize *P. vera* cultivars and wild *Pistacia* genotypes. Based on this study, the pistachio genome is about 600 Mb in size with a high heterozygosity rate that is probably owing to the dioecious mating system in this genus [10].

Most studies on *P. vera* have focused on limited SSR and random amplified polymorphic DNA (RAPD) markers development for germplasm characterization and sex determination [11–14], as well as the study of some physiological parameters in pistachio plants under drought and salt stresses [15–18]. However, there is no transcriptomic research and gene discovery at the large scale in this species, and in Anacardiaceae family only two RNA sequencing (RNA-seq) datasets are available from *Mangifera indica* [19] and *P. chinensis* [20]. In recent years, high-throughput RNA sequencing has emerged as an innovative and cost-effective tool for comprehensive transcriptome profiling in model and non-model plants [21]. While transcriptome analysis would be carried out using mapping-based approaches for model plants, deep sequencing followed by creating a high quality de novo assembled transcriptome is of first priority for downstream analysis in non-model plants [22, 23]. Most de novo transcriptome assemblers attempt to recognize the contiguous sequences by generating a de-Bruijn graph, which the k-mer length is one of the key factors that affect the assembly output. The optimal k-mer length for a given assembly relies on sequencing depth and genome/transcriptome complexity [21]. However, obtaining the high quality transcriptome assembly requires the assembly evaluation from multiple perspectives [24].

The objective of this study is to (1) create a robust pooled transcriptome assembly from leaf, stem, and root of *P. vera* under control and salinity conditions to provide a reference sequence, (2) compare the three well-known transcriptome assemblers, including CLC Genomics Workbench, Trinity and SOAPdenovo-Trans with single and multiple k-mer length using several length- and annotation-based metrics, (3) functionally annotate the reconstructed transcripts, (4) examine the biological pathways and genes related to salt stress, (5) develop the SSR markers for future marker-based studies, and (6) analyze selected salt-stress responsive genes using qRT-PCR.

The present study, as the first report on the transcriptome sequencing of *P. vera*, will not only provide plenty of informative data, but also paves the way for functional and comparative genomic studies on this species, as well as, other related species. Owing to similar signal transduction pathways and plant response mechanisms among salinity, cold, and drought stresses, the resulting genome-wide transcriptome map of pistachio under salt stress is a valuable platform for upcoming RNA-seq analysis of gene expression of this major species under these abiotic stresses. Finally, since root, stem and leaves of *P. vera* were sampled and sequenced at the high sequencing depth, the obtained annotated assembly will definitely facilitates RNA-seq analysis of any above tissues in future studies.

## Methods

### Plant materials and salinity treatment

In order to select the salt-sensitive and salt-tolerant pistachio cultivars, the surface-sterilized seeds of five cultivars, including *P. vera* L. cv. Sarakhs, Badami-zarand, Ghazvini, Akbari and Kaleghuchi as the main indigenous cultivars of Iran were obtained from Iranian Pistachio Research Institute, and grown in boxes containing Hoagland’s nutrient solution (pH = 5.8) with 16-h light/8-h dark photoperiods for 6 weeks. The 6-week-old plants were exposed to salt stress by adding 250 mM NaCl to the hydroponic culture medium. In order to avoid osmotic shock, salt was added incrementally up to the final salt concentration. The nutrient solution was renewed every 3 days. The experiment was performed with 30 plants per cultivar with three biological replicates. For cultivar selection, plant survival rate was measured after 8 days of exposure to salinity. Based on the results, Sarakhs and Ghazvini cultivars were determined as salt-sensitive and salt-tolerant cultivars with 24.4% and 65% of survival percentage after 8 days of salt stress, respectively (Additional file 1). For more assessment, sodium and potassium content as the main elements of salinity stress and Malondialdehyde (MDA), as the cell membrane lipid peroxidation index, were measured in roots of all cultivars on the 8th day after salt treatment. Based on statistical analysis, there were significant differences (*p*-value <0.05) among cultivars, specifically between Sarakhs and Ghazvini as we expected. The lowest level of sodium and the highest level of potassium were accumulated in the Ghazvini roots while the opposite pattern was observed for roots of Sarakhs. Similarly, in contrast to Sarakhs, the MDA content was the lowest in the Ghazvini roots (Additional file 1).

For transcriptome sequencing, the surface-sterilized seeds of selected rootstocks, *P. vera* L. cv. Sarakhs and Ghazvini as the most salt-sensitive and salt-tolerant cultivars, respectively, were grown and subjected to salt stress as above mentioned. We used 7 plants for each salt-sensitive and salt-tolerant cultivars with three biological replicates (in total, 21 plants for each cultivar) at each time point, including 0, 6, 24, and 48 h post salt treatment. The leaves, stems, and roots of 21 plants were separately harvested at each time point and immediately frozen in liquid nitrogen. For RNA extraction, we pooled 21 samples of each tissue at each time point together as one sample. Total RNA was finally isolated from 24 samples, containing two cultivars, three types of tissue, and four time points.

### RNA extraction, library preparation and RNA sequencing

The total RNA was separately isolated from 200 mg of harvested tissues (in total, 24 samples) using a modified CTAB (cetyltrimethyl ammonium bromide) method [25]. The purity and quantity of each RNA sample were determined by NanoDrop 2000 ™ micro-volume spectrophotometer (Thermo Scientific, Waltham, MA, USA), and gel electrophoresis. The final quality assessment was performed using Agilent Bio Analyzer 2100 prior to deep sequencing. Only RNA samples with RIN (RNA integrity number) of more than 8 were used for further processing. Extracted RNA samples were treated with RNase-free DNase I to remove the probable DNA contamination. Pooled RNA sample was made for comprehensive transcriptome sequencing through mixing an equal amount of extracted RNA (1 μg) from each sample. Library preparation from the pooled RNA sample representing 24 tissues of two pistachio cultivars under normal and stress conditions was performed as outlined in Illumina’s TruSeq Stranded mRNA Sample Prep Kit (Illumina Inc., U.S.A). The cDNA library was sequenced on the one lane of Illumina Hiseq 2000 platform as 2 × 100 run according to the manufacturer’s instructions. The raw sequencing reads produced in this study have been deposited at NCBI in the Short Read Archive (SRA) database under the accession number SRX1880621.

### Read pre-processing

Following the assessment of raw reads quality using FastQC tool (http://www.bioinformatics.babraham.ac.uk/projects/fastqc/), the reads were pre-processed to eliminate low quality bases and probable contaminants. We used CLC genomics workbench software (v7.5, CLC-Bio, Qiagen) for removing low quality bases (Phred score cutoff = 20) and adapter sequences. The probable ribosomal-derived reads were also filtered from the sequencing data using SortMeRNA tool prior to the assembly step [26].

### De novo transcriptome assembly

**Fig. 1:**
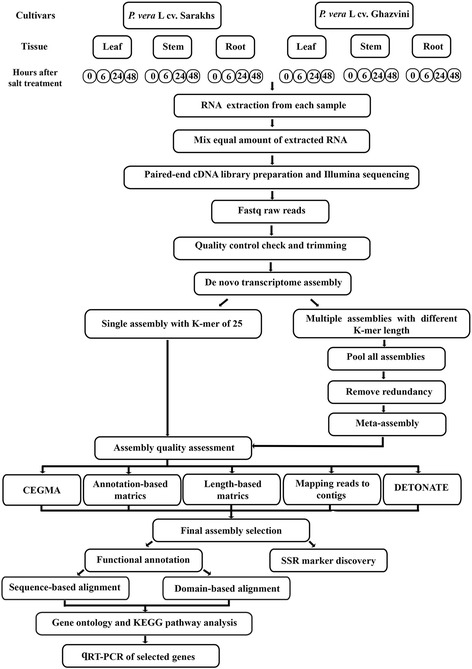
The workflow for pistachio transcriptome assembly and analysis. Two pistachio cultivars, salt-sensitive (Sarakhs) and salt-tolerant (Ghazvini), were selected. RNA was separately isolated from leaf, stem, and root of two cultivars after 0, 6, 24, and 48 h of salt treatment. Equal amount of extracted RNA was mixed to make a single pool for a deep sequencing on one lane of Illumina Hiseq 2000 as paired-end. Following quality control check and trimming, de novo assembly was carried out using Trinity, SOAPdenovo-Trans, and CLC genomics workbench softwares through two different strategies. After rigorous assembly quality assessment, final assembly was selected and exposed to functional annotation and SSR marker discover followed by gene ontology analysis and validation of some candidate genes by qRT-PCR

De novo transcriptome assembly was conducted using two strategies through three well-known assemblers, Trinity (v2014–07-17) [27], SOAPdenovo-Trans (v1.03) [28], and CLC genomics workbench (v7.5) (http://www.clcbio.com), which have been developed for the assembly of short reads by de Bruijn graph algorithm [29]. In the first strategy, clean reads were assembled to the contigs with k-mer of 25 using Trinity, SOAPdenovo-Trans and CLC programs with default settings except for Trinity, in which the strand-specific as well as in silico read normalization options were implemented. In the second strategy, different assemblies were made with the k-mer lengths of 25, 29, 33, 37, 41, 45, 49, 51, 55, 59, and 63 using SOAPdenovo-Trans and CLC tools; no-scaffold option was set during the assembly to avoid the strings of N’s connecting separate contigs. Contigs shorter than 300 bp were discarded from all the de Bruijn graph-based assemblies. All 9 individual k-mer assemblies resulted from SOAPdenovo-Trans and CLC tools were separately combined into a single merged assembly, which was further processed by CD-HIT-EST tool (v4.6.1–2012–08-2, 30] at 99% identity level (cd-hit-est. -c 0.99 -n 8 –r 1) in order to reduce redundancy and remove the identical fragments. The processed assembly was exposed to CAP3 program [30] with default settings to perform a meta-assembly and generate longer and more complete consensus sequences. The outputs of CAP3, contigs and singletons, were pooled together to form a final assembly. An overview of our workflow is illustrated in Fig. 1.

### Transcriptome assembly quality evaluation

The output of each assembly was interrogated using several quality metrics including, length- and annotation-based parameters. Contig length information such as N50 length, total contig number, average, and maximum contig length were assessed for each assembly using CLC software and ordinary Perl scripts. Raw reads were also aligned back to the each assembly by CLC program with the setting of mismatch/insertion/deletion costs at 2/3/3 and length fraction/similarity to 0.75/0.90 and the percentage of mapped back reads to transcripts were assessed. The completeness of each assembly was estimated using the Core Eukaryotic Genes Mapping Approach (CEGMA) tool [31]. Moreover, all protein sequences of *Citrus sinensis*, as the closely related species to *P. vera*, were downloaded from Phytozome (v10.3) and utilized for homology search. Each assembly was compared with *C. sinensis* proteome using BLASTX with the e-value cutoff of 1e-5 retaining only top match for each query sequence. Likewise, assembled transcripts were reverse annotated through the citrus dataset by TBLASTN with the same e-value cutoff using NCBI-BLAST software (v2.2.30+) [24]. Then, the number of unique proteins found in the BLASTX, the number of reciprocal best hits (RBH) and the ortholog hit ratio (OHR) were computed for each assembly as described by O’Neill and Emrich [24]. Further, the RSEM-EVAL tool from DETONATE (DE novo TranscriptOme rNaseq Assembly with or without the Truth Evaluation) package was employed for different assemblies assessment [32] (Fig. 1).

### Functional annotation of the assembled pistachio transcripts

Prior to annotation, TransDecoder program (v2.1.0) (https://transdecoder.github.io) was used in order to predict the protein-coding open reading frames (ORFs). To recognize homologous proteins, all deduced protein sequences resulted from Trinity assembly were aligned against the NR (non-redundant), UniProt (Swiss-Prot and TrEMBL), TAIR10 (the Arabidopsis information resource), and KOG (eukaryotic orthologous groups) protein databases using locally installed NCBI-BLAST software (v2.2.30+) with the e-value threshold of 1e-5. To annotate transcripts with GO terms, the best blast hit from the NR database was imported to Blast2GO software (v 3.2). Likewise, the predicted protein sequences were searched against Pfam, ProDom, ProSiteProfiles by InterProScan software (v5.14–53.0) [33] for retrieving conserved domains/motifs and corresponding GO terms. Additionally, KEGG (kyoto encyclopedia of genes and genomes) pathway analysis was conducted using the Automatic Annotation Server (KAAS; http://www.genome.jp/kegg/kaas/) with single-directional best hit (SBH) method for KEGG orthology (KO) assignments and pathway mapping.

### Candidate stress-responsive genes identification

To identify the potential stress-responsive genes in the *P. vera* transcriptome, all final assembled transcripts were searched against the genes involved in various abiotic stresses (salt, drought, and cold) [34, 35] using BLASTX with the e-value cutoff of 1e-5.

### Quantitative real-time PCR

The selected transcripts related to salinity-responsive genes were chosen for validation by quantitative real-time PCR (qRT-PCR). All required primers were designed using Oligo 7 software (Additional file 2); the elongation factor 1 alpha (*EF1α*) was applied as a reference gene [9]. qRT-PCR was carried out using SYBR Green real-time PCR master mix (Ampliqon, Denmark). The reaction mixture contained 1.0 μL of diluted cDNA sample, 0.5 μL of each of the forward and reverse primers (10 μM) and 10 μL real-time master mix with a final volume of 20 μL. The cycling conditions were as follows, initial activation of DNA polymerase at 95 °C for 10 min, followed by 40 cycles 94 °C for 30 s, 63 °C for 30 s and 72 °C for 20 s. Each sample was examined in three biological replications. A melting-curve analysis was 65 °C to 95 °C, with fluorescence measured every 0.5 °C at the end of each reaction to further confirm the specificity of the primer pairs. The relative gene expression was computed by Pfaffl formula [36] embedded in REST 2009 software.

### SSR marker discovery

The SSR Locator V.1 software was used for SSR identification, primer design and virtual PCR [37]. All type of SSRs, from dinucleotide to hexanucleotide repeats were searched on the transcriptome assembly generated by Trinity. The minimum repeat number of six for dinucleotide and five for all other repeats were specified for this analysis.

## Results and discussion

### Pistachio transcriptome sequencing

High-throughput sequencing technology has provided an excellent opportunity for transcriptome survey in non-model plant species, like *P. vera.* Here, a total of 394,579,946 million raw reads (90 Gb of data) as paired-end (PE) generated from a pooled RNA sample. For making a robust reference transcriptome that cover a wide range of transcripts, equal amount of extracted RNA from leaf, stem, and root of salt-tolerant and –sensitive cultivars at 0, 6, 24, and 48 h post salt treatment was pooled and sequenced on one lane of Illumina Hiseq 2000 platform. After trimming of the low quality bases and adapters as well as the removal of rRNA, a total of 368,953,262 million clean PE reads with an average quality score of 38 were reminded for transcriptome assembly (Table 1). rRNA is the most abundant transcript within total RNA, causing a major challenge during transcriptome assembly by decreasing the sequencing depth for informative mRNAs [38]. While we used poly A^+^ selection method for mRNA enrichment, 3.7% of reads were still aligned to ribosomal RNA that properly removed before doing de novo assembly. The resulting data and subsequent analyses constitute an unprecedented look into the *P. vera* transcriptome at a single nucleotide resolution.

**Table 1:** Basic statistics of pistachio sequencing reads obtained from Illumina HiSeq-2000

Parameter	Value
Number of raw reads	394,579,946
Reads average length before trimming (bp)	100
Reads average length after trimming (bp)	98
Number of ribosomal RNA reads	14,333,680
Number of reads after trimming	368,953,262
Average quality score	38
GC content (%)	44

### Generation of a de novo-assembled, genome-wide transcriptome map of *P. vera* and assembly statistics

In order to construct a high quality assembly, several transcriptome assemblies were created using three state-of-the-art de Bruijn graph-based assemblers, including CLC Genomics Workbench, SOAPdenovo-Trans, and Trinity, which accept multiple k-mer and single k-mer value, respectively. As Trinity efficiently produced a transcriptome assembly with its default k-mer of 25, we selected this k-mer length across three assemblers to compare them at the single k-mer size (the first strategy). While the contig number, length distribution, and length-weighted medians (N50 and N90) were comparable between assemblies produced by SOAPdenovo-Trans and CLC softwares, the highest number of contigs (144,103), of which 59,727 (41.4%) were at least 1 kb in length, were generated by Trinity (Table 2). With the constant k-mer of 25, a considerable difference was found in average contig length and N50 value among assemblies. Again, the highest average of transcript length (1139 bp) and N50 size (1679 bp) were obtained from Trinity assembly (Table 2). Due to the non-uniform nature of transcriptome assembly, low and high abundant transcripts might theoretically represent at smaller and larger k-mer size, respectively [39]. Therefore, in the second strategy, we used the k-mer size of 25–63 with the step size of 4 for making multiple k-mer assemblies through SOAPdenovo-Trans and CLC. All generated contigs by two assemblers with various k-mer lengths were independently merged and fed to cd-hit-est. program for removing redundant contigs. This tool is a popular clustering program based on the greedy incremental clustering method, which groups nucleotide sequences of a given dataset into clusters that meet a user-defined similarity threshold and the longest sequences of each cluster are selected as representatives [40]. This step significantly reduced the contig total number from 602,146 to 165,895 for SOAPdenovo-Trans assembly and 584,686 to 169,120 for CLC one. The non-redundant contigs were then subjected to CAP3 software to compute the overlaps and produce a consensus assembly. CAP3 is an efficient sequence assembler that connects highly similar overlapping sequences and thus further reduces transcript redundancy [30]. The resulting contigs and singletons obtained from CAP3 were collapsed together for generating a final merged assembly. As shown in Table 2, the contig average length and N50 value were notably increased in the merged assemblies compared to the corresponding single assemblies (Table 2). These results are consistent with previous reports of the higher performance of merged assemblies in *Crocus sativus* [41].

**Table 2:** Transcriptome assembly quality evaluation metrics

Parameters	CLC	SOAPdenovo-Trans	Trinity	Merged assembly (SOAPdenovo-Trans)	Merged assembly (CLC)
Number of contigs	83,390	85,091	144,103	93,865	90,632
Average transcript length (bp)	787	695	1139	885	945
Minimum transcript length (bp)	300	300	300	300	300
Maximum transcript length (bp)	12,370	12,097	13,939	13,329	12,167
N50 (bp)	909	788	1679	1300	1450
N90 (bp)	392	361	489	397	425
Percentage of contig ≥1 kb	20.92	16.47	41.44	30%	33.83%
Percentage of mapped back reads to assembly	88.51	83.81	97.49	93.54	96.98
Percentage of mapped reads in pairs	78.23	70.71	89.93	83.67	87.76
Percentage of mapped broken paired reads	10.28	13.1	7.86	9.54	9.22
Percentage of complete cores proteins by CEGMA analysis	79.03	69.35	97.98	95.16	94.35
Percentage of partial cores proteins by CEGMA analysis	93.15	93.15	99.6	97.74	98.79
The number of unique proteins found in blastx	20,782	20,872	25,065	20,789	20,911
The number of unique contigs hit by proteins in the tblastn	44,479	44,503	44,807	44,302	44,803
The number of unique contigs with reciprocal best hits	13,730	13,724	15,690	14,003	14,101
The number of unique contigs with orthologue hit ratio of 0.8–1	5963	7249	12,903	7750	8423
RSEM-EVAL score	−19,590,755,435	−22,457,309,826	−11,702,493,371	−14,998,853,445	−13,665,328,361

Transcriptome assembly evaluation metrics for single 25 k-mer assemblies generated by CLC genomics workbench, SOAPdenovo-Trans and Trinity as well as the merged assembly with k-mer length ranging from 25 to 63 with the step size of 4

### Assessing transcriptome assembly quality

Although parameters like, N50 and average contig length are commonly used statistics for genome assembly evaluation, they may have little informative value for transcriptome assembly evaluation [42]. In fact, N50 measures the continuity of contigs but not their accuracy. Similarly, the larger N50 length or smaller contig number do not necessarily imply a better de novo transcriptome assembly [24, 43]. Hence, in addition to assessing the basic length-based parameters, the assembly quality was evaluated using a number of more stringent criteria as explained below (Fig. 1).

### Mapping reads to the assembled transcripts

The percentage of reads mapped back to assembled transcripts (RMBT) is considered to examine the assembly completeness, implicating the read inclusion amount to construct the assembly [44]. Ideally, the high quality assembly should have the high RMBT percentage. The high RMBT in our study was obtained from the Trinity and merged assemblies, followed by the single k-mer assembly created by CLC and the assembly made by SOAPdenovo-Trans that had the lowest mapped reads (Table 2). Our results indicate that Trinity at the single k-mer length utilizes the majority of sequencing reads to construct the assembly, in contrast to other programs and strategies. Additionally, as compared with other assemblies, Trinity assembly had the highest percentage of mapped reads in pairs and the lowest percentage of broken paired reads, the paired reads that each mate of a read mapped on two different assembled transcripts (Table 2), which may implicate the lower fragmented transcripts produced by Trinity.

### Identification of widely conserved eukaryotic genes

CEGMA program conducts a similarity search of assembly against the subset of KOG database consisting 248 highly conserved proteins from a wide range of eukaryotes [31]. These proteins mostly belong to housekeeping genes group and were expected to be expressed in many tissues [45]. We incorporated it in our pipeline for evaluating the assembly completeness, the number of proteins represented in each assembly is shown in Table 2. CEGMA can also discriminate between full-length (complete) and partial predicted core proteins. While CEGMA analysis recognized 243 out of 248 core proteins (97.98%) as complete (70% alignment length of assembled transcript with core protein) within the Trinity assembly, the 79.03 and 69.35% of full-length core proteins were detected for the single k-mer assembly created by CLC and SOAPdenovo-Trans softwares, respectively (Table 2). Comparing to these single k-mer assemblies, the merged assemblies covered the more number of core proteins, referring to the higher transcript number came from this approach. The percentage of partial core proteins was higher than complete ones in all cases (Table 2), offering some genes were split on the several contigs.

### Comparisons with orthologs

**Fig. 2:**
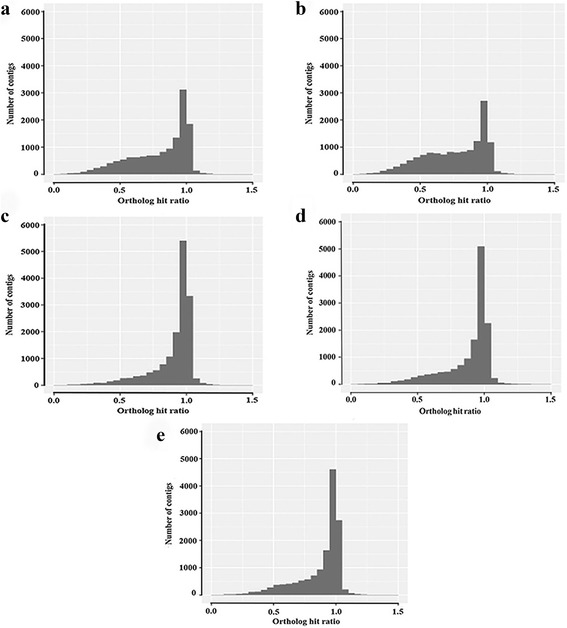
Ortholog hit ratio of assembled transcripts for various assemblies. **a** Single assembly, CLC genomics workbench. **b** Single assembly, SOAPdenovo-Trans. **c** Single assembly, Trinity. **d** Merged assembly, CLC genomics workbench. **e** Merged assembly, SOAPdenovo-trans

Moreover, we used several annotation-based metrics for evaluating the transcriptome assembly quality as described by O’Neil and Emrich [24]. For this assessment, BLASTX and TBLASTN algorithms were applied for comparison of the assembled transcriptome with *Citrus sinensis* proteome, as the closest reference species. According to a phylogenetic study of mango (*M. indica*) chloroplast DNA, *C. sinensis* was closely related to *M. indica* [46], which is belongs to the Anacardiaceae family. As presented in Table 2, the number of unique *C. sinensis* proteins found in the BLASTX search was comparable for all assemblies, except for the Trinity assembly with the highest percentage of citrus proteins (Table 2). Overall, about 45–54% of *C. sinensis* proteins were covered by each assembly, probably due to species divergence and the lack of complete gene expression in our experimental conditions. To identify the reciprocal best hit (RBH) and actual ortholog between *P. vera* and *C. sinensis*, reverse annotation was performed using TBLASTN. The number of RBH was computed based on the BLASTX and TBLASTN outputs for each assembly. As a result, the highest number of RBH (15,690) was produced by Trinity assembly followed by merged and single assemblies (Table 2). It is worth noting that the RBH level depends on the evolutionary divergence between the species of interest and related reference [47]. Considering the fact that the high quality transcriptome assembly would reconstruct more full-length transcripts, the OHR was also estimated for the various assemblies to determine the contig content assembled into an ortholog sequence [48, 49]. Since assessing transcript integrity relies on the contig and its best hit are ortholog, only contigs with RBH were used for OHR calculation. Therefore this metric is conservative as parts of orthologs may be represented by contigs that are not the reciprocal best hit [50]. Our findings showed that the highest percentage of full length transcripts was constructed by Trinity, so that the ortholog hit ratio for 12,904 contigs (82.24%) with RBH was 0.8–1, referring to near full length or full length transcript (Table 2). This parameter was computed by comparing the length of assembled contigs relative to the length of known ortholog sequences, hence the OHR of 1 considered as a full length transcript [24]. As the OHR distribution plot illustrates (Fig. 2), Trinity assembly followed by the merged assemblies produced significantly more full-length transcripts than the single assemblies generated by SOAPdenovo-Trans and CLC tools. However, gaining partial transcripts with OHR of less than 0.8 represented by all assemblies was unavoidable, implying that the full-length transcript construction is a challenge during de novo transcriptome assembly, particularly for non-model organisms. Although, the length-based parameters were significantly improved in the merged assembly as compared with the single assembly, the number of unique proteins found in BLASTX and the number of unique contigs with reciprocal best hits were slightly better in these assemblies. Hence, using the annotation-based metrics together with the length-based parameters allows an improved assessment of the transcriptome assembly quality, compared to the solely use of the length-based parameters.

### Assembly evaluation using DETONATE

RSEM-EVAL is a reference-free approach bundled with DETONATE package that was recently developed for transcriptome assembly evaluation [32]. In this study, RSEM-EVAL score was calculated for each transcriptome assembly as well. This score is based on a probabilistic model that accepts the clean reads and assembly as input [51]. Assemblies with the higher score are considered to be better. As presented in Table 2, the RSEM-EVAL score of merged assemblies generated by CLC and SOAPdenovo-Trans softwares were slightly different, but higher than the corresponding single assemblies. Among all assemblies, Trinity assembly with the highest score was the best one (Table 2), revealing that this program performed more accurately on individual contigs level than CLC and SOAPdenovo-trans. Overall, according to all above mentioned evaluation criteria, we adopted Trinity output for further analyses.

### Functional annotation of *P. vera* transcriptome

**Fig. 3:**
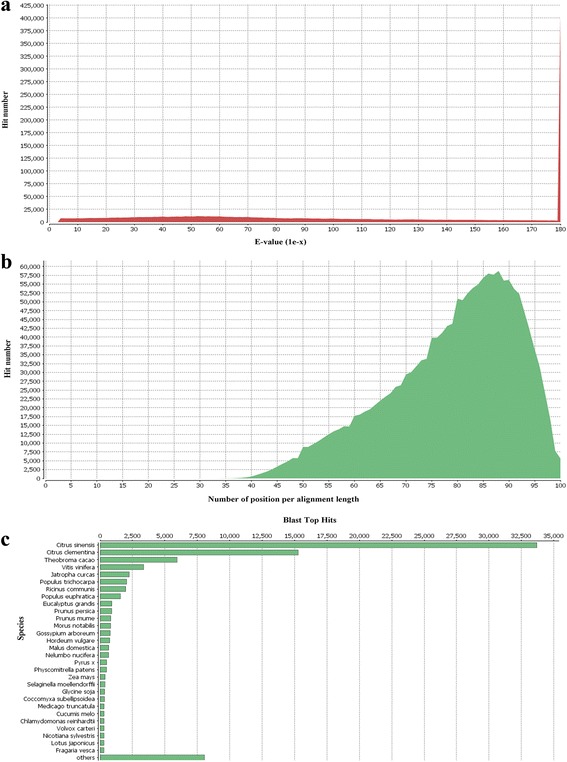
Graphical representations of functional annotations in *P. vera* transcriptome. **a** E-value distribution graph. **b** Similarity distribution graph. **c** Top-hit species distribution graph

The integration of transcriptome assembly with functional annotation is the main aspect of mining transcriptomic data in a non-model organism. Here, to predict the function of assembled pistachio transcripts, similarity search was performed at two levels, sequence-based and domain-based alignments. To facilitate the procedure, transcripts with open reading frames (ORF) were extracted from the Trinity assembly using TransDecoder program. Out of 144,103 transcripts, 94,826 (65.8%) potential protein sequences were obtained, of which 52,971 (55.86%) sequence were reported as full length (Table 3). For sequence-based annotation, all deduced protein sequences were aligned against the NR database using BLASTP algorithm and the best blast hit imported to Blast2GO software for downstream analysis. Out of 94,826 sequences, 84,117 (88.7%) had a significant blast hit with the e-value threshold of 1e-5 (Table 3 and Additional file 3). Although we applied an e-value cutoff of 1e-5 for homology search analysis, the e-value for the major fraction of matched sequences ranged between 1e-10 and 1e-180 as illustrated in Fig. 3a. Similarity distribution demonstrated that the maximum number of matched sequences have 88% positive alignment length (Fig. 3b), the lack of best blast hits with less than 35% sequence similarity with respect to query implied the appropriate homology with the query. In terms of species distribution, the majority of annotated sequences showed the highest homology to sequences from *Citrus sinensis* (39.7%), followed by *Citrus clementina* (18%), *Theobroma cacao* (7%) and *Vitis vinifera* (4%) (Fig. 3c). It has been reported that *M. indica* and *C. sinensis* are phylogenetically related species [46]. *P. vera* and *M. indica,* belong to the Anacardiaceae family, suggesting that the pistachio transcriptome achieved in the present study was properly annotated. Meanwhile, only 161 sequences in our assembly showed best matches with *M. indica,* which could be due to little publicly available sequence information for this species. In order to obtain further descriptions and improve the pistachio annotation, the current assembly was blasted against two other protein databases, including UniProt (Swiss-Prot and TrEMBL) and TAIR10. Compared to UniProt, a total of 82,180 (86.6%) significant best blast hits were returned with the e-value threshold of 1e-5 (Table 3). In the case of TAIR10, comprising a complete reference genome and well-annotated sequences for *Arabidopsis thaliana*, 73,276 (77.27%) sequences displayed significant homology with this database (Table 3).

**Table 3:** Functional annotation summary of *P. vera* transcriptome

Parameter	Number of sequence
Potential protein sequences	94,826
Annotated sequence against NR	84,117
Annotated sequence against UniProt	82,180
Annotated sequence against TAIR10	73,276
Annotated sequence against InterProScan	66,029
Annotated sequence against KOG	33,288
Sequences matching all five databases	29,119
Sequence annotated with GO terms	68,539
Sequences assigned with EC numbers	21,598
Annotated sequence against KEGG	34,924

Considering that the proteins with little similarity at the sequence level can share conserved domains, we carried out a domain-based annotation by searching the candidate protein sequences against Pfam, ProDom, and ProSiteProfiles databases using InterProScan program. Results revealed that a total of 66,029 sequences (69.77%) were categorized into 4235 domains/families. Based on the number of *P. vera* transcripts contained in each InterProScan domains/families, the InterPro domains/families ranked and listed the top 30 abundant domains/families in Table 4. The most represented protein region was a Pentatricopeptide repeat (PPR) (IPR002885) with 14,926 assigned sequences. Consistent with our findings, it has been reported that proteins with PPR site constitute one of the largest protein families in plants with 450 members in *Arabidopsis thaliana* and more than 600 members in *Oryza sativa* [52, 53]. PPR proteins are mostly targeted to mitochondria or chloroplasts, where they are involved in organelle gene expression via RNA processing, splicing, stability, editing, translation, and plant response to abiotic stresses [52, 54]. Other commonly occurring domains/sites in the *P. vera* transcriptome were protein kinase domains (IPR000719), leucine-rich repeats (IPR001611), and WD40 repeats (IPR001680). Protein kinase domain and its subclass, serine-threonine/tyrosine-protein kinase were recognized to regulate the majority of cellular processes, including signal transduction, differentiation, as well as, cell growth and development. Leucine-rich repeat regions are known to have a repeating stretch of 20–29 amino acids that form an α/β horseshoe fold involved in the formation of protein-protein interactions [55]. Similarly, WD40 repeat act as a site for protein-protein interaction and WD40-containing proteins serve as platforms for the assembly of protein complexes or mediators of transient interplay among other proteins [56].

**Table 4:** List of the top-hit 30 InterPro domains in *P.vera* transcriptome

InterPro domain	Description	Number of sequence
IPR002885	Pentatricopeptide repeat	14,926
IPR000719	Protein kinase domain	50,94
IPR001611	Leucine-rich repeat	3983
IPR001680	WD40 repeat	2556
IPR000504	RNA recognition motif domain	1828
IPR019734	Tetratricopeptide repeat	1235
IPR002048	EF-hand domain	1185
IPR001245	Serine-threonine/tyrosine-protein kinase catalytic domain	1176
IPR001841	Zinc finger, RING-type	1155
IPR020683	Ankyrin repeat-containing domain	1055
IPR017986	WD40-repeat-containing domain	9,30
IPR002110	Ankyrin repeat	883
IPR018108	Mitochondrial substrate/solute carrier	839
IPR001650	Helicase, C-terminal	808
IPR003439	ABC transporter-like	719
IPR001128	Cytochrome P450	692
IPR002182	NB-ARC	632
IPR001810	F-box domain	562
IPR013026	Tetratricopeptide repeat-containing domain	538
IPR000571	Zinc finger, CCCH-type	517
IPR001932	PPM-type phosphatase domain	508
IPR017930	Myb domain	493
IPR011598	Myc-type, basic helix-loop-helix (bHLH) domain	489
IPR000048	ITE - IQ motif, EF-hand binding site	488
IPR005123	Oxoglutarate/iron-dependent dioxygenase	484
IPR011527	ABC transporter type 1, transmembrane domain	455
IPR013210	Leucine-rich repeat-containing N-terminal, plant-type	444
IPR000008	C2 domain	442
IPR001623	DnaJ domain	428
IPR014001	Helicase superfamily 1/2, ATP-binding domain	420

**Fig. 4:**
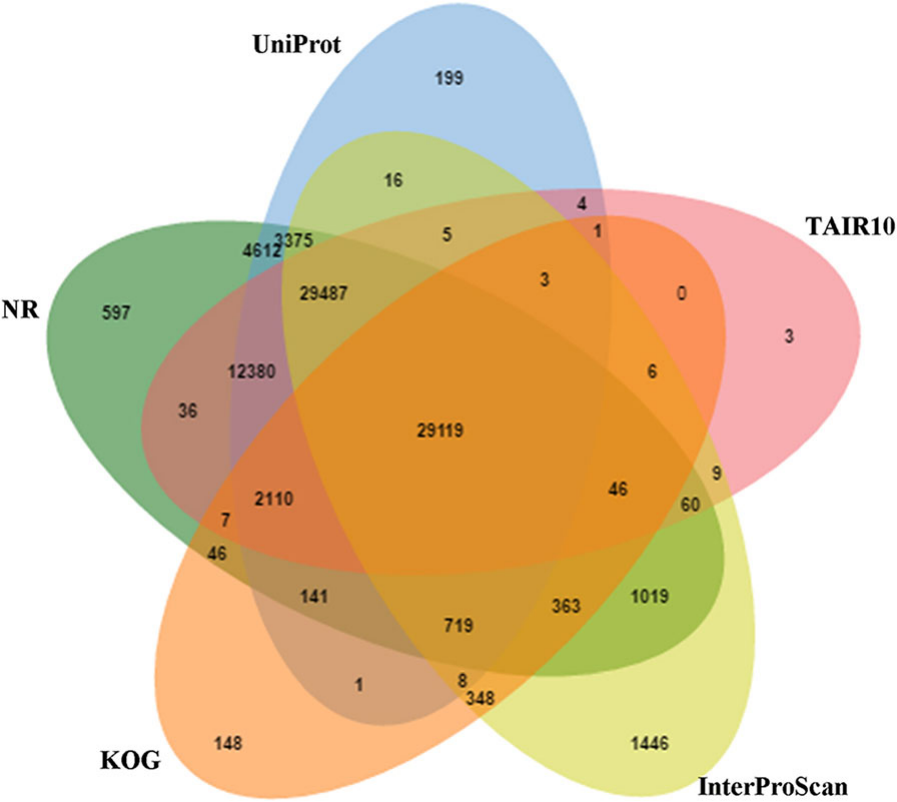
Venn diagram shows the BLAST results of *P. vera* against five databases, including NR, UniProt, KOG, TAIR10, and InterProScan. The number of transcripts with significant hits is presented in each intersection of the Venn diagram

The KOG database built on phylogenetic classification of proteins encoded in 66 complete genomes, including bacteria, plants, and animals. Each KOG cluster consists of a protein or a group of proteins from at least 3 different eukaryotic lineages further clustered based on their function [57]. We aligned the translated assembly to the KOG database in order to predict and classify their possible functions. KOG classification of our assembled transcripts revealed that 33,288 protein sequences were clustered into 25 functional categories under four larger groups (metabolism, cellular processes and signaling, information storage and processing, and poorly characterized) (Additional file 4). Among the different KOG categories, the highest number of sequences were assigned to the “signal transduction mechanisms” category (4902, 14.7%), followed by “general function prediction only” (4108, 12.34%) and “posttranslational modification, protein turnover, chaperones” (3832, 11.5%). In the metabolism group, “Carbohydrate transport and metabolism” (5.9%), “amino acid transport and metabolism” (5.4%), and “Intracellular trafficking, secretion, and vesicular transport” (5.3%) were also highly represented. However, 1748 (5.3%) of total protein sequences annotated with KOG database were classified as “function unknown”, suggesting that *P. vera* is almost a phylogenetically distant species compared to those present in the KOG database. Taken together, 29,119 sequences exhibited the similarity to proteins in all of the five public databases, including NR, UniProt, TAIR10, KOG and InterProScan (Fig. 4).

### Gene ontology and KEGG pathway classification

**Fig. 5:**
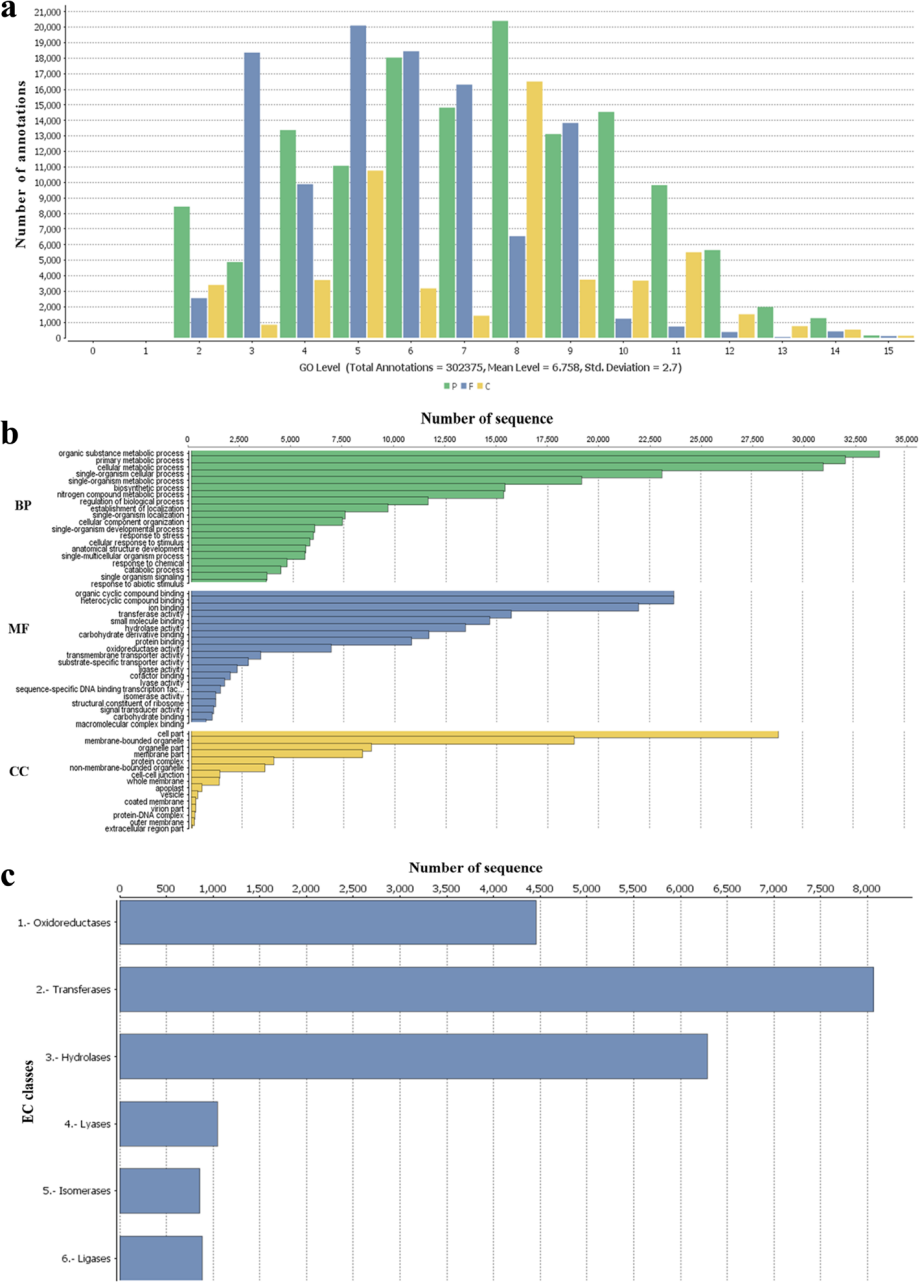
GO and enzyme classification of assembled pistachio transcripts. **a** GO level distribution of annotated sequences. **b** GO classification across three main categories. Biological process (BP), Molecular Function (MF), and Cellular Component (CC).**c** Catalytic activity distribution in annotated *P. vera* transcriptome

**Fig. 6:**
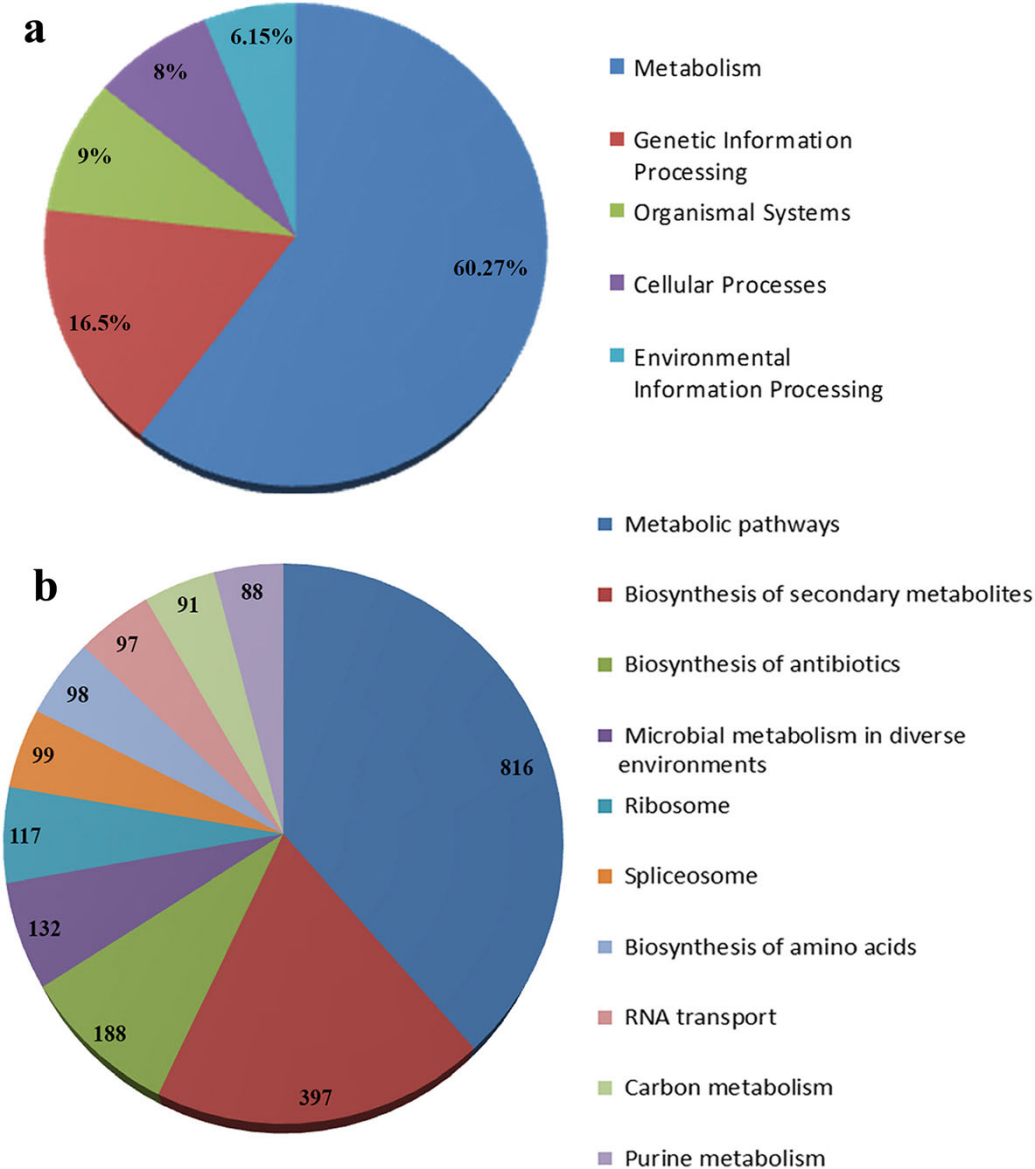
KEGG classification of pistachio transcriptome. **a** KEGG distribution of annotated transcripts into biological categories. **b** The top 10 pathways with the highest transcript numbers

Gene ontology (GO) is an international classification system that provides standardized vocabulary for description of genes and their products functions in all organisms [58]. Here, to functionally categorize the pistachio transcripts, the best blast hits retrieved from the NR database was imported into Blast2GO software to capture GO terms and enzyme code numbers. Results specified that the maximum number of GO terms was achieved by UniProtKB database and TAIR. Out of 84,117 sequences with NR annotation, a total of 68,539 (80.76%) were associated with 302,375 GO terms (Fig. 5a), which classified into the three GO categories: biological process, molecular function, and cellular component. The majority of GO terms were assigned to the biological process (13,7628, 45.52%), followed by molecular function (109,033, 36.06%), and cellular component (55,714, 18.43%). We also surveyed the distribution of pistachio GO terms across the major sub-categories (third level) as illustrated in Fig. 5b. For the biological process category, genes involved in the “organic substance metabolic process”, “primary metabolic process”, and “cellular metabolic process” were highly represented. The most enriched GO terms in the molecular function category were “organic cyclic compound binding”, “heterocyclic compound binding”, and “ion binding”. For the cellular component, transcripts with the most frequent GO terms being “cell”, “cell part”, and “organelle”. Accordingly, the annotated sequences involved in the main GO classification regulate the basic biological and metabolic processes, which were consistent with the most representative GO terms in the mango leaf and fruit transcriptome [19, 46]. Since a portion of our transcriptome assembly was generated from plants under salt treatment, stress- and signaling-linked annotation were frequently found in this dataset. A total of 1753 sequences fell under 19 stress-related GO terms, of which “response to salt stress” and “response to oxidative stress” were the main terms, constituting 37.2% and 21.5% of sequences, respectively. Similarly, 4009 sequences were distributed into 91 signaling pathway-related terms. The high frequency of GO terms associated with stress and signaling confirmed the sampling under salinity stress and properly making the transcriptome assembly. Of the 68,539 sequences associated with GO terms, a total of 21,598 (31.5%) sequences were determined as enzyme. Among various enzyme types, transferases (37.35%), followed by hydrolases (29.1%) and oxidoreductases (20.62%) were the most abundant enzymes (Fig. 5c). A large number of annotated enzymes in the present assembly proposes the presence of transcripts involved in various biological pathways [59, 60]. Therefore, in order to fully recognize the active biological pathways in the *P. vera* transcriptome, the potential protein sequences were searched against the KEGG database using KAAS server with the single-directional best hit method and the corresponding KEGG orthology (KO) identifiers assigned to the sequences. KEGG is a highly integrated database providing information on biological systems and their relationships at the molecular, cellular and organism levels [61]. In total, 34,924 sequences were found to be involved in 279 KEGG pathways, covering five main KEGG categories, including metabolism, genetic information processing, organismal systems, cellular processes and environmental information processing (Fig. 6a) The metabolic pathway (816 members), biosynthesis of secondary metabolites (397 members) and the biosynthesis of antibiotics (188 members) were the most representative pathways by the unique sequences. The top 10 pathways with the most assigned sequences were summarized in Fig. 6b and the entire functional KEGG pathway classification of the *P. vera* transcriptome have been shown in Additional file 5. We believe that our findings will provide precious resources for investigating specific processes and functions in pistachio and other woody plants.

### Identification of transcription factors

**Fig. 7:**
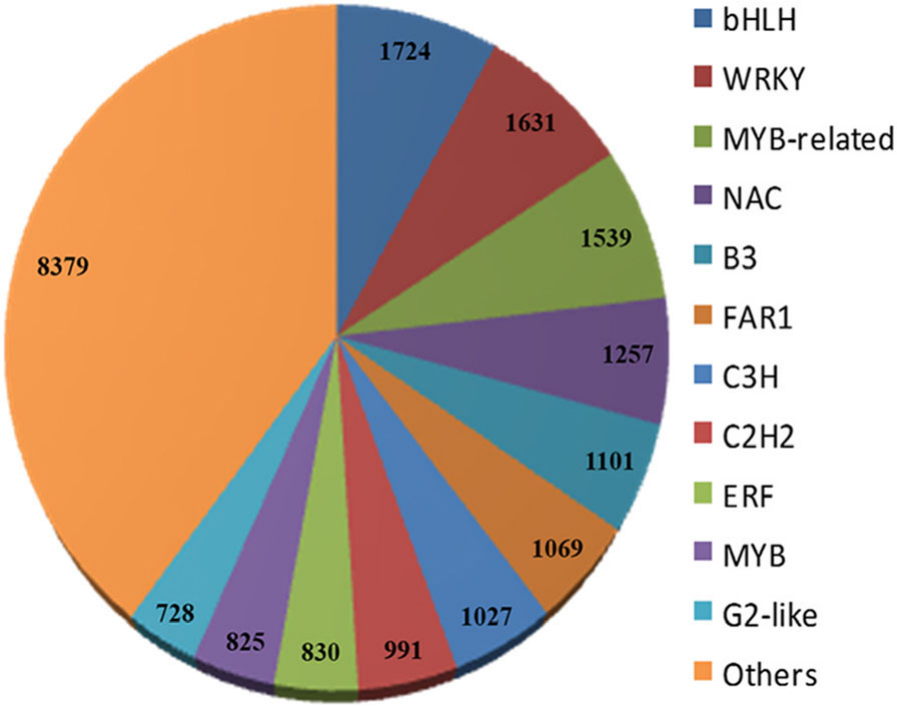
Distribution of *P. vera* transcripts into different transcription factor families

Under abiotic stresses, including salinity, many transcription factors (TF) operate to convert the stress-induced signals to plant cellular responses [62, 63]. Over the past decades, numerous abiotic stress-related TFs have been determined in the various plants, but nothing has been studied in pistachio tree so far. As a result of homology search of *P. vera* transcriptome against the plant transcription factor database using BLASTX, a total of 21,099 transcripts were annotated and categorized into 57 families (Fig. 7). Of the various transcription factor families, *bHLH, WRKY, MYB*-related, and *NAC* were the four most abundant families with more than 1000 assigned transcripts while *NZZ/SP* and *LFY* had only one member (Additional file 6). Recent extensive genetic and molecular studies have proved that plenty of transcription factors belonging to the *NAC, MYB, MYB*-related, *WRKY, bHLH*, and *bZIP* families play central roles in plant responses to abiotic and biotic stresses [64–68]. Given that the activation of a large number of stress-responsive genes is mediated through specific TFs, identification of potential transcription factors will improve our understanding of the effective molecular mechanisms of abiotic stress tolerance in pistachio in future studies.

### Salt stress-related pathways and genes survey

Investigating the biological pathways play a key role in gaining insight into the advanced genomics researches. Plants response to abiotic stresses, including salinity is highly complex that involves various pathways, such as carbohydrate metabolism, biosynthesis of secondary metabolites, plant hormones biosynthesis and signal transduction pathways [69].

### Transcripts related to plant hormones

Plant hormones trigger particular signaling cascades upon sensing the abiotic stress signals, which eventually results in an improved growth pattern under adverse conditions. The KEGG pathway mapping showed that the pistachio transcriptome assembly captures almost all key components of signal transduction pathways of all plant hormones, including auxin, cytokinin, gibberellin, abscisic acid (ABA), ethylene, brassinosteroid, jasmonic acid, and salicylic acid (Additional file 7). The prominent role of ABA during the plant salt response led us to examine ABA biosynthesis and its signal transduction pathway within the pistachio dataset. Through our KEGG analysis, we discover the main ABA biosynthesis pathway genes, including beta-carotene 3-hydroxylase (*CRTz*), beta-ring hydroxylase (*LUT5*), zeaxanthin epoxidase (*ZEP*), 9-*cis*-epoxycarotenoid dioxygenase (*NCED*) and ABA-aldehyde (*ABA2*) in the *P. vera* dataset (Additional file 8a). Moreover, the enzymes of ABA 8′-hydroxylase (EC:1.14.13.93) required for ABA catabolism and ABA beta-glucosyltransferase (EC:2.4.1.263) involved in ABA inactivation by conjugation with glucose were also identified in our assembly. However, ABA function is required to hormone sense and transduce the signal to the cellular components. Here, we could detect all ABA signaling components such as the ABA receptor (*PYR1*), *SnRK2* protein kinase, protein phosphatase of *PP2C* and ABA responsive element binding factor (*ABF*), which regulates the expression of ABA-responsive genes (Additional file 8b). Based on our results, most of the transcripts had multiple copies. For instance, the numbers of transcripts encoding *ZEP, NCED, PYR1*, and *PP2C* were 27, 4, 5, and 33, respectively.

Auxin is another multi-functional hormone that is responsible not only for normal plant growth and development, but also for modulating plant growth under stress conditions. Besides, it interacts with ABA, enhancing plant sensitivity to the ABA effect on root growth inhibition under osmotic stress [70]. Auxin biosynthesis initiates from the shikimate pathway, leading to the synthesis of aromatic amino acids, like Tryptophan (Trp), a known precursor of auxin biosynthesis [71]. Additionally, the shikimate pathway is one of the crucial routes for primary and secondary metabolism in plants, which also generates precursors for the biosynthesis of different indole compounds, alkaloids, and other aromatic metabolites, lignin, as well as flavonoids. In our transcriptome dataset, all genes encoding for enzymes involved in the shikimate pathway were identified (Additional file 9a). Shikimate pathway-derived Trp converts into indole-3-acetic acid (IAA) through a two-step reaction that is catalyzed by aminotransferase of Arabidopsis (*TAA*) and YUCCA (*YUC)* family of flavin monooxygenases [71]. This auxin biosynthesis pathway is a major route for auxin production, which is conserved among plants, including pistachio as our data analysis revealed the corresponding transcripts. Furthermore, the *P. vera* transcriptome dataset contained enzymes like indoleacetaldoxime dehydratase (EC: 4.99.1.6), cytochrome P450 monooxygenase (EC:1.14.-.-), amidase (EC:3.5.1.4), and indole-3-acetaldehyde oxidase (EC:1.2.3.7) that contributed to the auxin biosynthesis from alternative pathways (Additional file 9b). In addition to these key biosynthetic genes, all genes encoding proteins in the auxin signal transduction pathway, auxin influx carrier (*AUX1*), transport inhibitor response1 (*TIR1*), *Aux/IAA* repressor protein, *ARF* transcription factor*, GH3*, and *SAUR* were found in the pistachio transcriptome dataset (Additional file 9c). Considering the major role of ABA in plant abiotic stresses response and its interaction with auxin, our findings offer valuable information to decipher the ABA-regulated processes and discover the probable stress response mechanisms in pistachio.

### Transcripts related to flavonoid biosynthesis

**Fig. 8:**
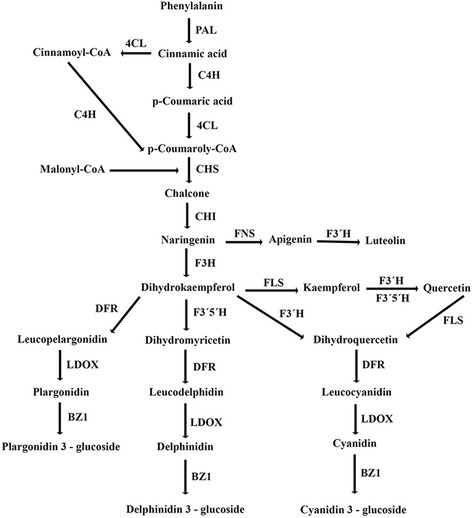
The proposed pathway for the biosynthesis of major flavonoids, including luteolin, quercetin, kaempferol and various types of anthocyanins (pelargonidin, delphinidin, and cyanidin) in pistachio. Phenylalanine ammonia-lyase (*PAL*), 4-coumarate CoA ligase (*4CL*), Trans-cinnamate 4-monooxygenase (*C4H*), Chalcone synthase (*CHS*), Chalcone isomerase (*CHI*), Flavone synthase (*FNS*), Flavonoid-3′-hydroxylase (*F3’H*), Flavone-3-hydroxylase (F3H), Flavonol synthase (*FLS*), Flavonoid 3′5′-hydroxylase (*F3’5’H*), Dihydroflavonol-4-reductase (*DFR*), Leucoanthocyanidin dioxygenase (*LDOX*), Anthocyanidin 3-O-glucosyltransferase (*BZ1*). The genes listed in this Fig. were all identified in *P. vera* transcriptome

Flavonoids constitute a major group of polyphenolic secondary metabolites with a vast array of biological functions in plants, including stress protection. They operate as an antioxidant defense system for protecting plants against oxidative damage originating from various abiotic and biotic stresses [72, 73]. The biosynthesis of flavonoids requires the enzymes involved in the phenylpropanoids pathway and its flavonoid branch pathways. In the present study, KEGG analysis of the *P. vera* transcriptome showed the presence of 18 transcripts to be contributed to the biosynthesis of different compounds of phenylpropanoid pathway (Additional file 10). In our dataset, starting from initial enzymes of flavonoids biosynthesis (through the phenylpropanoid pathway), such as phenylalanine ammonia lyase (PAL) (EC: 4.3.1.24), cinnamate 4- monooxygenase (EC: 1.14.13.11), 4-coumarate CoA ligase (EC: 6.2.1.12), and chalcone synthase (EC: 2.3.1.74) were recognized. PAL, the key enzyme in the phenolic biosynthesis pathway, is generally considered as a biochemical marker of stress conditions. It has been reported that the PAL enzymatic activity is different across various *P. vera* cultivars and there is a positive correlation among the PAL activity, phenolic compounds and the higher tolerance of pistachio cultivar against environmental stresses [74]. Here, ten transcripts encoding *PAL* were identified in the pistachio transcriptome that could be potentially used as molecular markers for selecting the salt-tolerant pistachio species. Similarly, it has been recently proved that the chalcone synthase, at both transcript and protein levels, was positively related to salinity tolerance in the salt-tolerant soybean genotypes [75], indicating the pivotal role of flavonoids biosynthesis in salinity tolerance. Further, chalcone isomerase (EC: 5.5.1.6) that catalyzes chalcone isomerisation into naringenin was identified in this dataset. Naringenin is a central branch point for the synthesis of several major groups of flavonoids, including flavanones, flavonols, and anthocyanins [76]. Interestingly, all transcripts encoding the enzymes participated in the biosynthesis of the main class of flavonoids were discovered in the present transcriptome (Additional file 11). We proposed the biosynthesis pathway for major flavonoids in pistachio in Fig. 8. Briefly, flavonoids are synthesized via the phenylpropanoid pathway where phenylalanine is converted to coumaroyl-CoA through a series of enzymatic modifications. Coumaroyl-CoA is then combined with malonyl-CoA to yield chalcone, which catalyzed by chalcone synthase, and isomerized into naringenin via chalcone isomerase activity. The metabolic pathway continues through a series of enzymatic reactions to yield flavanones, dihydroflavonols, and anthocyanins. Flavonoids of quercetin, luteolin, myricetin, and kaempferol have been detected in the leaf extracts of pistachio through high-performance liquid chromatography (HPLC) analysis. In the case of anthocyanins, only two types of cyanidin (cyanidin 3-galactoside and cyanidin 3-glucoside) were experimentally reported in the pistachio nut [77]. However, detecting the active biological pathways related to other anthocyanin types may imply the presence of corresponding anthocyanins, including delphinidin and pelargonidin in pistachio (Fig. 8).

### Transcripts related to stress-responsive genes

Salt stress tolerance is considered a quantitative trait, requiring the contribution of several genes for plant survival under adverse conditions. While a large number of stress-responsive genes were determined in various plant species, there is no report for pistachio that can prevent the understanding of stress tolerance mechanism in this major salt-tolerant tree. Due to similar signal transduction pathways and plant responses among abiotic stresses, like salinity, drought and cold, a BLASTX analysis (e-value cutoff of 1e-5) against a database made from above-mentioned stresses-responsive genes (see Methods) were conducted and 13,097 transcripts as candidate stress-associated genes identified. The current pistachio transcriptome assembly represented 617 out of the 704 abiotic stress-related proteins, suggesting our assembly enough enriched with genes involved in the various aspects of salt, drought and cold stresses.

The products of stress-inducible genes can be classified into two major groups; the first group is comprised of functional proteins, like late embryogenesis abundant (LEA) proteins, osmotin and chaperones and the second group contains the regulatory proteins, such as transcription factors, protein kinases and protein phosphatases that involved in the regulation of stress-responsive genes expression and signaling pathways [78]. Here, 116 *LEA*, 9 osmotin, and 160 chaperone transcripts were recognized in the pistachio transcriptome assembly. The expression of a large number of stress-responsive genes is regulated by the mitogen-activated protein kinase (MAPK) cascade. The MAPK signaling pathway consists of three components, MAPK kinase kinase (MAPKKK), MAPK kinase (MAPKK) and MAPK that play a pivotal role in transferring the extracellular signals to the nucleus [79]. In the current transcriptome assembly, 332 transcripts encoding the various members of MAPK pathway responding to abiotic stresses were identified. Among them, *MAPK4* and *MAPK6* with 230 transcripts were the most abundant transcripts, which is associated with their function during salt stress. The both *MAPK4* and *MAPK6* are positively involved in the plant abiotic stresses (salt, drought, and cold); Arabidopsis overexpressors *MAPK4* and *MAPK6,* constitutively expressed stress-induced genes, resulting in an increased salinity and freezing tolerance [80]. One of the downstream targets of MAPK6 is a plasma membrane Na^+^/H^+^ antiporter (SOS1) [81], which mediates Na^+^ efflux from the roots and loading of Na^+^ ions in the xylem. The salt overly sensitive (SOS) pathway that comprises SOS1, SOS2, and SOS3 has emerged as a main mechanism to maintain ion homeostasis in plants under salt stress. Salinity evokes a cytosolic calcium signal that is perceived by SOS3 and activates SOS2, a serine/threonine protein kinase. The SOS2-SOS3 complex regulates the expression level and activity of SOS1 [82]. Our pistachio transcriptome assembly represented all components of this pathway, so that 11, 7, and 16 transcripts encoding *SOS1*, *SOS2*, and *SOS3*, respectively, were recognized. Therefore, the SOS pathway is conserved in pistachio tree and presumably conferred salt tolerance in salt-tolerant pistachio species. In the case of salt stress signaling, in addition to SOS3 family, calcium-dependent protein kinase (CDPK) and calcineurin B-like protein-interacting protein kinase (CIPK) have emerged to be the main proteins in linking of stress signal to specific protein phosphorylation cascades [83]. We also characterized 94 stress-responsive transcripts corresponding to calcium-dependent protein kinase (*CDPK*) and 133 transcripts encoding calcineurin B-like protein-interacting protein kinase (*CIPK*) in the present assembly.

One of the common consequences of abiotic stresses is the increased reactive oxygen species (ROS) content [84]. Although ROS are considered as the signaling molecules with the regulatory role during plant development and abiotic stress responses, plants have evolved enzymatic and non-enzymatic antioxidants to maintain the steady-state level of ROS and alleviate the oxidative stress [85]. Considering the central role of enzymatic antioxidants, the assembled transcriptome was surveyed for stress-responsive transcripts encoding this type of enzymes. As a result, 24 transcripts for superoxide-dismutase (*SOD*), 88 glutathione S-transferase and 204 peroxidase family transcripts were identified. In the case of *SOD*, *Cu-Zn SOD* was the main isoform that correlated with its broad localization in cytosol, apoplast, peroxisomes and chloroplasts.

### Gene validation and expression analysis

**Fig. 9:**
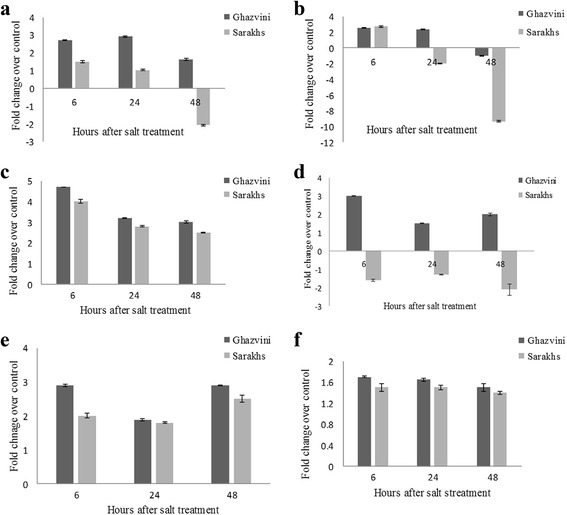
Fold change of selected salt-stress responsive genes over three time points of salt stress in root of *P. vera* L. cv Sarakhs and Ghazvini. The stressed samples were quantified to the non-stressed. *EF1α* was used as a reference gene for data normalization. Mean value and standard deviation (SD) were presented for three biological replicates. **a** *ZEP*, **b** *NCED3*, **c** *PP2CA*, **d** *SOS1*, **e** *dehydrin*, **f** *CDPK11*

In order to verify that the reconstructed transcripts were expressed, several key stress-responsive genes, including *ZEP* and *NCED3* contributing to ABA biosynthesis along with *PP2CA* to be involved in the ABA signaling pathway, as well as *NHX7*, sodium/proton antiporter known as *SOS1*, dehydrin, a member of group 2 LEA and *CDPK11* were selected to perform qRT-PCR analysis in root samples of *P. vera* L. cv. Ghazvini and Sarakhs after 0, 6, 24, and 48 h of salt treatment. All primers pair generated a single band with the expected size on the gene electrophoresis and one peak on the melting curve, implying that the assembled transcripts were reliable and subsequent gene expression profiling were conceivable. Based on our results, the expression of *ZEP* gene was up-regulated up to 48 h of salt treatment in the salt-tolerant cultivar (Ghazvini) while the slightly up-regulation by 1.25 fold until 24 h and the significant down-regulation by −2.1 fold after 48 h of salinity were observed in the salt-sensitive cultivar (Sarakhs) (Fig. 9a).

The transcript level of *NCED3* was found to be higher in the salt-tolerant than salt-sensitive cultivar. As shown in Fig. 9b, the abundance of *NCED3* transcript was increased at the 6 and 24 h and slightly decreased (fold change of −0.98) after 48 h exposure to stress in the salt-tolerant cultivar. But, in the salt-sensitive cultivar, *NCED3* expression level was enhanced only over the 6 h of the treatment, then considerably reduction by −9 fold occurred at the 48 h of salinity.

ZEP and NCED are the key enzymes in the ABA biosynthesis pathway that contribute to convert the zeaxanthin to violaxanthin and epoxycarotenoids to xanthoxin, respectively [86]. In agreement with previous studies that revealed the elevated expression of *ZEP* and *NCED3* under salt stress in *Arabiopsis thaliana* [83] and *Populus tomentosa* [87], we found the similar trend, especially for salt-tolerant cultivar in the present study. Considering the positive correlation between the increased level of these transcripts and ABA content, higher salinity tolerance of Ghazvini as compared to Sarakhs can probably result from the more ABA production under salt treatment. In accordance with these results, the more ABA accumulation in *P. vera* L. cv. Badami (another salt-tolerant cultivar) than *P. vera* L. cv. Sarakhs under salt treatment has been reported by Panahi (2009) [88]. The more ABA content in the salt-tolerant maize [89] and tomato [90] under salinity has been previously demonstrated. The expression analysis of *PP2C*, one of the main genes in ABA signaling pathway revealed that its transcript level was enhanced in both cultivars, Ghazvini and Sarakhs, during salinity stress. As shown in Fig. 9c, this up-regulation is slightly but significantly higher in Ghazvini than Sarakhs. The protein phosphatase of PP2CA, as a negative regulator, mediates plant responses to salt stress by regulation of ABA signaling pathway. *PP2CA* gene is highly induced by salinity and ABA as shown by previous researches in *Oryza sativa* [91] and Chrysanthemum [92]. From the *PP2C* expression pattern, like other plants, a transcriptional negative feedback mechanism may regulate the ABA response in the both pistachio cultivars under salinity treatment.

*SOS1*, one of the central components of SOS pathway, exhibited the contrasted expression pattern between salt-tolerant and salt-sensitive pistachio cultivars (Fig. 9d). The transcript level of *SOS1* was significantly reduced over all three time points in Sarakhs, whereas its abundance was increased after 24 and 48 h of salinity in Ghazvini. Interestingly, when compared with Sarakhs, we detected a lower accumulation of Na^+^ in roots of Ghazvini (unpublished data), which may correlate with the up-regulation of *SOS1* in the salt-tolerant cultivar. Therefore, pistachio salinity tolerance may partly refer to the active SOS pathway and sodium extrusion from cytoplasm. The similar result has been previously reported in salt-tolerant cotton genotype*, Gossypium hirsutum* [93].

As Fig. 9e and f show, in the both cultivars, expression of *dehydrin* and *CDPK11* were induced at all time points of salt stress. However, the genes up-regulation is slightly higher in salt-tolerant cultivar than salt-sensitive cultivar. In consistent with our results, the up-regulation of both genes in response to drought and high salinity stresses has been proved [93, 94]. Overall, the expression analysis confirmed that the selected stress-responsive genes are expressed at higher level in tolerant cultivar than sensitive one in response to salt treatment. However, further gene expression analysis at the large scale is required to exactly decipher the pistachio salt tolerance mechanism.

### Characterization of simple sequence repeats (SSRs)

SSRs are one of the most informative and versatile molecular markers, which are commonly used in genetic diversity evaluation, conservation genomics and genetic mapping studies [95]. In order to identify and conserve the salt-tolerant pistachio rootstocks, which is classified as a near threatened by IUCN (International Union for Conservation of Nature), the development of SSR markers is highly desirable. Therefore, we screened all contigs resulted from Trinity to discover potential SSRs for future researches. In total, 11,337 SSRs defined as di-to hexanucleotide motifs were recognized in 11,130 contigs, with 204 contigs bearing more than one SSR. Di- and trinucleotide repeats were the most abundant SSRs, accounting for 40–44% of total SSRs, followed by tetra- (9.5%), panta- (3.1%) and hexanucleotide repeats (2.2%) (Table 5), which is consistent with other plants [96–98]. The pistachio transcriptome was rich in GA/TC (12.13%), AG/CT (11.02%), AT/AT (8.32%), TA/TA (8.04%), GAA/TTC (5%) and AGA/TCT (4.02%). To make these SSR markers useful, Primer 3 was applied to design primer pairs for each SSR. A total of 7605 primer pairs were generated from the microsatellites with sufficient flanking sequences. All primer pairs were submitted to virtual PCR within SSR Locator tool that 2702 of them produced a single amplicon, suggesting the specificity of the corresponding SSR marker (Additional file 12). It has been reported that SSR markers that generate one in silico PCR product should be the putative single-locus markers and could be especially useful [98]. This large-scale marker discovery facilitates future researches for finding the salt tolerance-related markers and conserving the tolerant pistachio cultivars.

**Table 5:** Statistics of SSRs in the *P.vera* transcriptome

Total number of sequences	144,103
Total number of SSRs	11,337
Number of SSR containing sequences	11,130
Number of dinucleotide repeats	5063 (44.7%)
Number of trinucleotide repeats	4605 (40.6%)
Number of tetranucleotide repeats	1075 (9.5%)
Number of pentanucleotide repeats	350 (3.1%)
Number of hexanucleotide repeats	244 (2.2%)

## Conclusion

The present study is the first attempt at investigating the whole transcriptome sequencing of *P. vera*. A total of 368,953,262 million clean PE reads were created from various tissues of two pistachio cultivars with contrasting salinity tolerance under the control and salinity conditions by Illumina Hiseq 2000 platform. Following the comprehensive comparison of different assemblers using multiple length- and annotation-based metrics, the Trinity assembly was selected and functionally annotated against several protein databases. A total of 29,119 sequences received the best hit with known homologous proteins in all five used protein databases. The highest number of transcripts has been assigned to the NAC, MYB, MYB-related, WRKY, bHLH, and bZIP transcription factors families, consistent with their critical roles in plant salt stress responses. Most genes involved in plant hormone biosynthesis and signaling pathways as well as pathways to be contributed to secondary metabolite biosynthesis were found in pistachio transcriptome assembly. We also propose the biosynthetic pathway of major flavonoids in pistachio. A total of 13,097 transcripts found to be the stress-responsive and some of them validated by qRT-PCR, further confirm the accuracy of assembly. Further, 11,337 SSRs with a frequency of 14.4 kb were identified, which the most abundant being dinucleotide repeats. Taken together, given the usage of various pistachio tissues (leaf, stem, and root) and the similarity in signaling pathways and plant responses during salt, drought, and cold stresses, the current research could provide a valuable foundation for future RNA-seq analysis of *P. vera* under any of these stresses to discover the potential molecular mechanisms of abiotic stresses response and accelerate the breeding of new cultivars with more tolerance against abiotic stresses.

## Additional files

Additional file 1: Plant survival rate, Na^+^ and K^+^ level and Malondialdehyde (MDA) concentration parameters used for selecting the salt-sensitive and salt-tolerant cultivars. (DOCX 220 kb)

Additional file 2: Primer sequence used for qRT-PCR analysis. (DOCX 15 kb)

Additional file 3: Top BLAST hits from NCBI NR database with corresponding GO terms, Enzyme code and InterPro IDs. (XLSX 5822 kb)

Additional file 4: Eukaryotic orthologous groups (KOG) functional classification of pistachio transcriptome**.** A) RNA processing and modification; B) Chromatin structure and dynamics; C) Energy production and conversion; D) Cell cycle control, cell division, chromosome partitioning; E) Amino acid transport and metabolism; F) Nucleotide transport and metabolism; G) Carbohydrate transport and metabolism; H) Coenzyme transport and metabolism; I) Lipid transport and metabolism; J) Translation, ribosomal structure and biogenesis; K) Transcription; L) Replication, recombination and repair; M) Cell wall/membrane/envelope biogenesis; N) Cell motility; O) Post-translational modification, protein turnover, chaperones; P) Inorganic ion transport and metabolism; Q) Secondary metabolites biosynthesis, transport and catabolism; R) General function prediction only; S) Function unknown; T) Signal transduction mechanisms; U) Intracellular trafficking, secretion, and vesicular transport; V) Defense mechanisms; W) Extracellular structures; Y) Nuclear structure; Z) Cytoskeleton. (JPEG 84 kb)

Additional file 5: KEGG pathway classification of *P. vera* transcriptome (XLSX 19 kb)

Additional file 6: The reconstructed pistachio sequences that shows significant homology with the transcription factors (XLSX 10 kb)

Additional file 7: Plant hormone signal transduction pathway. The ortholog genes in pistachio indicated by green color. (JPEG 618 kb)

Additional file 8: Genes involved in the carotenoid biosynthesis in the pistachio transcriptome as shown by pink highlighting. a) ABA biosynthesis pathway. b) ABA signal transduction pathway. (JPEG 1096 kb)

Additional file 9: Genes involved in the shikimate biosynthesis a), auxin biosynthesis b), and auxin signal transduction pathways c) within pistachio transcriptome, as indicated by pink color. (JPEG 1494 kb)

Additional file 10: Phenylpropanoid biosynthesis pathway. The ortholog genes in pistachio transcriptome indicated by green highlighting. (JPEG 704 kb)

Additional file 11: Flavonoid biosynthesis pathway. The ortholog genes in pistachio transcriptome specified by green highlighting. (JPEG 608 kb)

Additional file 12: Primer pairs was used for virtual PCR to amplify SSR repeats. (XLSX 395 kb)

## Abbreviations

ABA: Abscisic acid
CEGMA: Core eukaryotic genes mapping approach
CTAB: Cetyltrimethyl ammonium bromide
DETONATE: De novo transcriptome rnaseq assembly with or without the truth evaluation
*EF1α*: Elongation factor 1 alpha
EST: Expressed sequence tag
GO: Gene ontology
HPLC: High-performance liquid chromatography
KEGG: Kyoto encyclopedia of genes and genomes
KO: KEGG orthology
KOG: Eukaryotic orthologous groups
LEA: Late embryogenesis abundant
MAPK: Mitogen-activated protein kinase
MDA: Malondialdehyde
NCBI: National center for biotechnology information
OHR: Ortholog hit ratio
PE: Paired-end
PPR: Pentatricopeptide repeat
qRT-PCR: Quantitative real-time PCR
RAPD: Random amplified polymorphic DNA
RBH: Reciprocal best hits
RIN: RNA integrity number
RMBT: Reads mapped back to assembled transcripts
RNA-seq: RNA sequencing
SBH: Single-directional best hit
SOS: Salt overly sensitive
SSR: Simple sequence repeats
TAIR: Arabidopsis information resource
TF: Transcription factors
Trp: Tryptophan
