# Supplementary material for: A genome-wide transcriptome map of pistachio (Pistacia vera L.) provides novel insights into salinity-related genes and marker discovery
Source: BMC Genomics. 2017 Aug 17;18:627. doi: 10.1186/s12864-017-3989-7 (PMC5559799; doi:10.1186/s12864-017-3989-7)
Supplement: Supplementary file 1 — Plant survival rate, Na+ and K+ level and Malondialdehyde (MDA) concentration parameters used for selecting the salt-sensitive and salt-tolerant cultivars. (DOCX 220 kb) [file 12864_2017_3989_MOESM1_ESM.docx]

**Additional file 1**. For salt-sensitive and salt-tolerant cultivar selection, plant survival rate (Fig.1), Na+ and K+ level (Figs.2 and 3) and Malondialdehyde (MDA) concentration (Fig.4) were measured after 8 days of exposure to salinity.

**Fig. 1** Survival percentage on 8^th^ day after salt stress. Sarakhs and Ghazvini cultivars with 24.4% and 65% of survival percentage were determined as salt-sensitive and salt-tolerant cultivars, respectively.

**Fig. 2** Sodium content in the roots of genotypes on 8^th^ day after salinity. Ghazvini and Sarakhs accumulated the lowest and hisghet sodium in their roots, respectively

**Fig. 3** Potassium content in the roots of genotypes on 8^th^ day after salinity. Ghazvini and Sarakhs accumulated the highest and lowest of potassium in their roots, respectively.

**Fig. 4** MDA level in the roots of genotypes on the 8^th^ day after salinity. The lowest MDA level was observed in Ghazvini and Badami-zarand and the highest level of MDA was detected in Sarakhs and Kaleghuchi cultivars.
