# Supplementary material for: A genome-wide transcriptome map of pistachio (Pistacia vera L.) provides novel insights into salinity-related genes and marker discovery
Source: BMC Genomics. 2017 Aug 17;18:627. doi: 10.1186/s12864-017-3989-7 (PMC5559799; doi:10.1186/s12864-017-3989-7)
Supplement: Supplementary file 2 — Primer sequence used for qRT-PCR analysis. (DOCX 15 kb) [file 12864_2017_3989_MOESM2_ESM.docx]

| Primer name | sequences (5'-3') | Tm (°C) | PCR product length (bp) |
| --- | --- | --- | --- |
| F-PP2C | GGAGACGGAGAGAGATGGAAGAC | 61 | 217 |
| R-PP2C | CGATCCCACTCTTCAGCTATCAC |  |  |
| F-CDPK | CATGGCCCAACATATCAGACAG | 60 | 112 |
| R-CDPK | CCACAATCCAGGGGTGACATA |  |  |
| F-NHX7 | GAGGCATTTGGTGATCTAGGAGAGG | 61 | 218 |
| R-NHX7 | TCGTCAAGCATTTCCCAGTAAGCA |  |  |
| F-dehydrin | GCGAGCAGAAAGGGCTGTA | 60 | 148 |
| R-dehyrin | TGCTGCTTCACGCCATCAC |  |  |
| F-ZEP | ACTTTGCAACAAATCCTCGCT | 61 | 171 |
| R-ZEP | CCTCACCTTCGACCATATTCCAT |  |  |
| F-NCED | CAACGCCGGACTGGTTTACT | 60 | 178 |
| R-NCED | TTTGCCCGAAACAGGATCAAC |  |  |
| F-EF1α | GGCAAGGTATGATGAAATCGTG | 63 | 123 |
| R-EF1α | ATCACCCTCAAATCCAGAGATG |  |  |

**Additional file 2. Primer sequence used for RT-PCR analysis**
