# Supplementary figures and images for: A genome-wide transcriptome map of pistachio (Pistacia vera L.) provides novel insights into salinity-related genes and marker discovery
Source: BMC Genomics. 2017 Aug 17;18:627. doi: 10.1186/s12864-017-3989-7 (PMC5559799; doi:10.1186/s12864-017-3989-7)

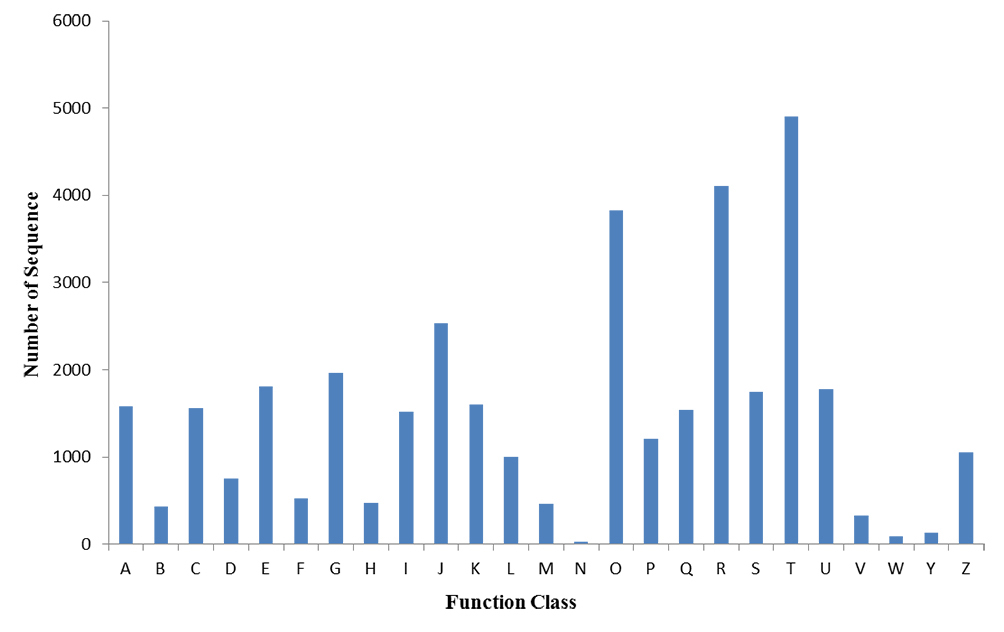

Supplement: Supplementary file 4 — Eukaryotic orthologous groups (KOG) functional classification of pistachio transcriptome. A) RNA processing and modification; B) Chromatin structure and dynamics; C) Energy production and conversion; D) Cell cycle control, cell division, chromosome partitioning; E) Amino acid transport and metabolism; F) Nucleotide transport and metabolism; G) Carbohydrate transport and metabolism; H) Coenzyme transport and metabolism; I) Lipid transport and metabolism; J) Translation, ribosomal structure and biogenesis; K) Transcription; L) Replication, recombination and repair; M) Cell wall/membrane/envelope biogenesis; N) Cell motility; O) Post-translational modification, protein turnover, chaperones; P) Inorganic ion transport and metabolism; Q) Secondary metabolites biosynthesis, transport and catabolism; R) General function prediction only; S) Function unknown; T) Signal transduction mechanisms; U) Intracellular trafficking, secretion, and vesicular transport; V) Defense mechanisms; W) Extracellular structures; Y) Nuclear structure; Z) Cytoskeleton. (JPEG 84 kb) [file 12864_2017_3989_MOESM4_ESM.jpg]

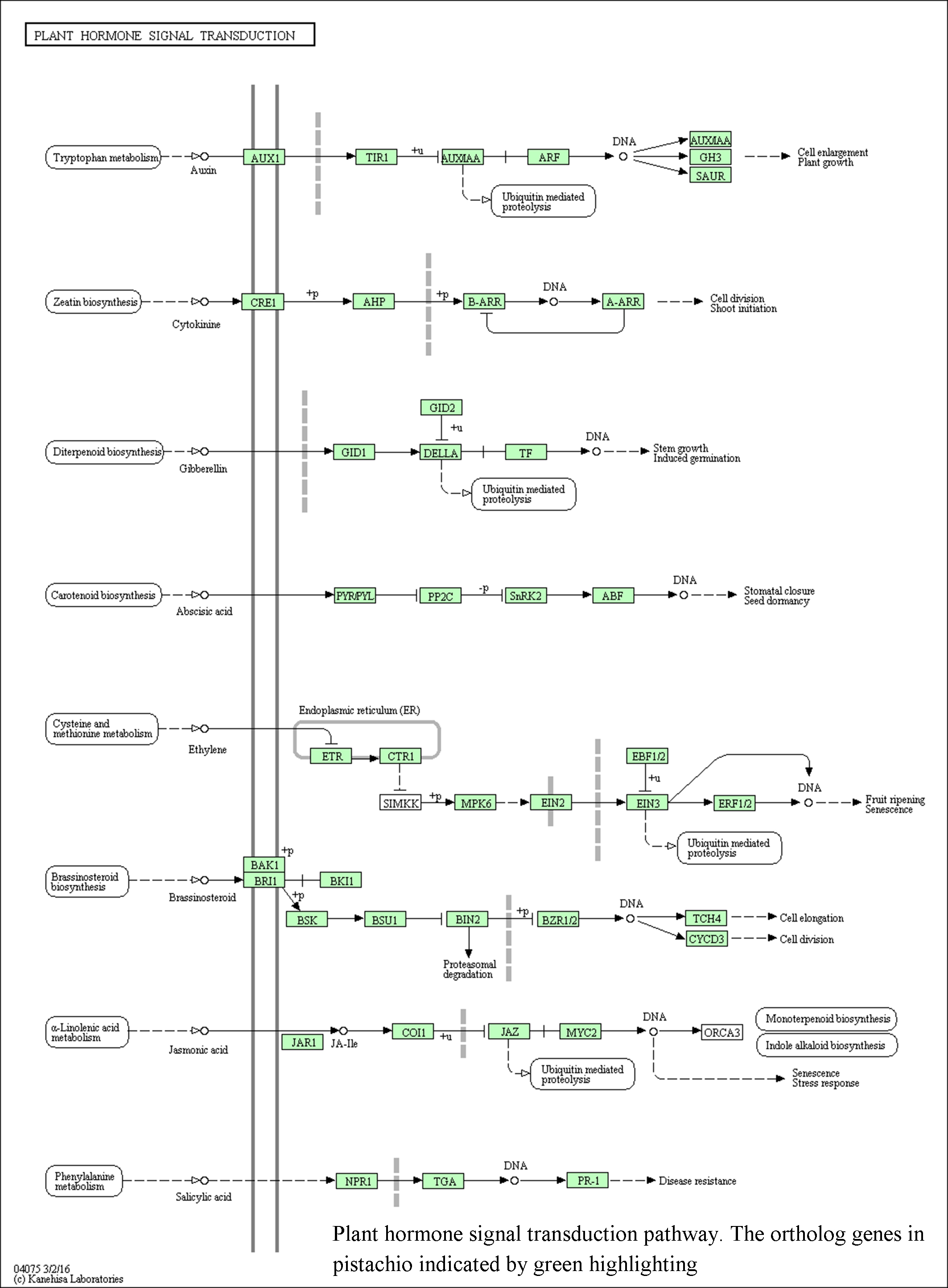

Supplement: Supplementary file 7 — Plant hormone signal transduction pathway. The ortholog genes in pistachio indicated by green color. (JPEG 618 kb) [file 12864_2017_3989_MOESM7_ESM.jpg]

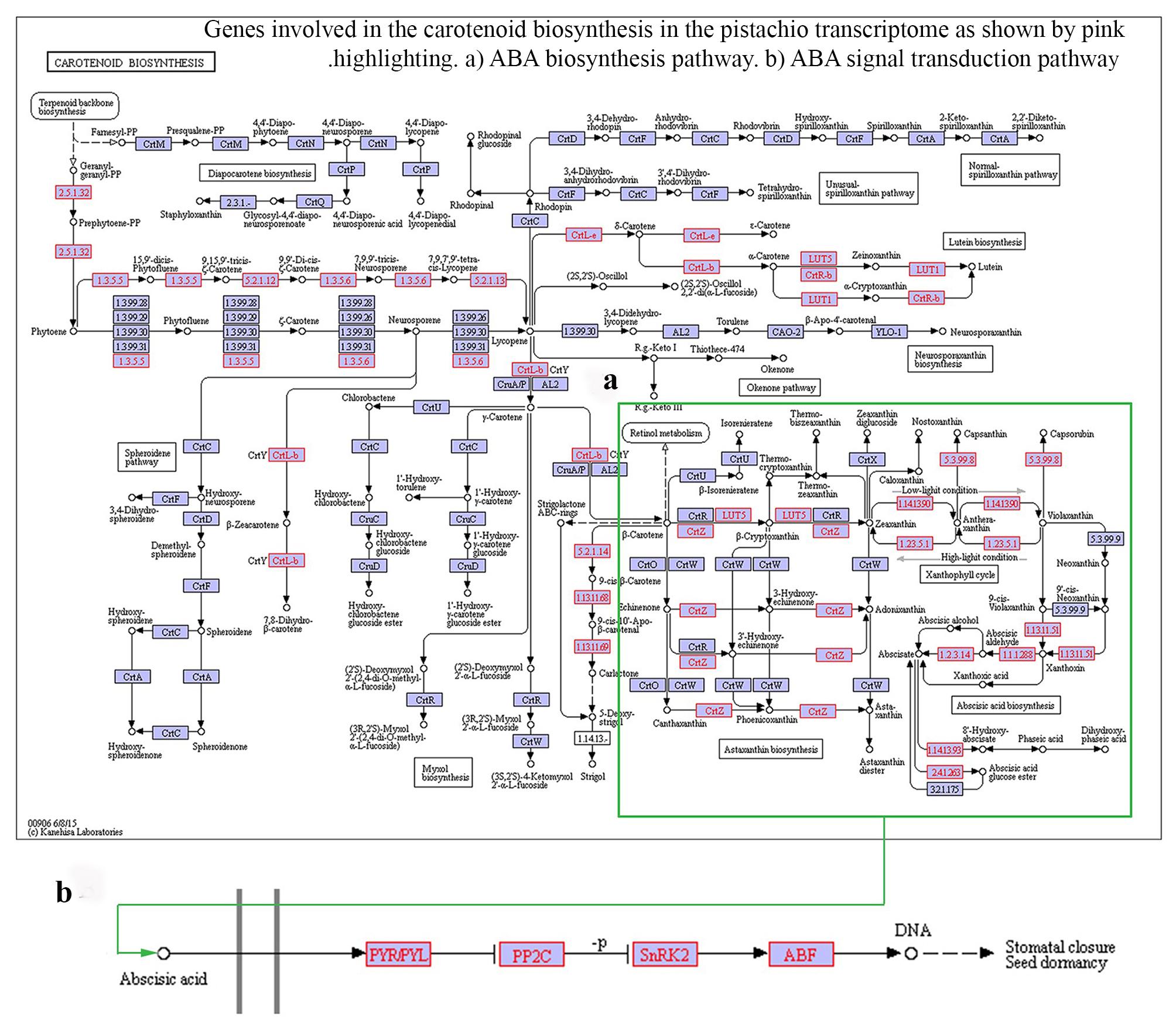

Supplement: Supplementary file 8 — Genes involved in the carotenoid biosynthesis in the pistachio transcriptome as shown by pink highlighting. a) ABA biosynthesis pathway. b) ABA signal transduction pathway. (JPEG 1096 kb) [file 12864_2017_3989_MOESM8_ESM.jpg]

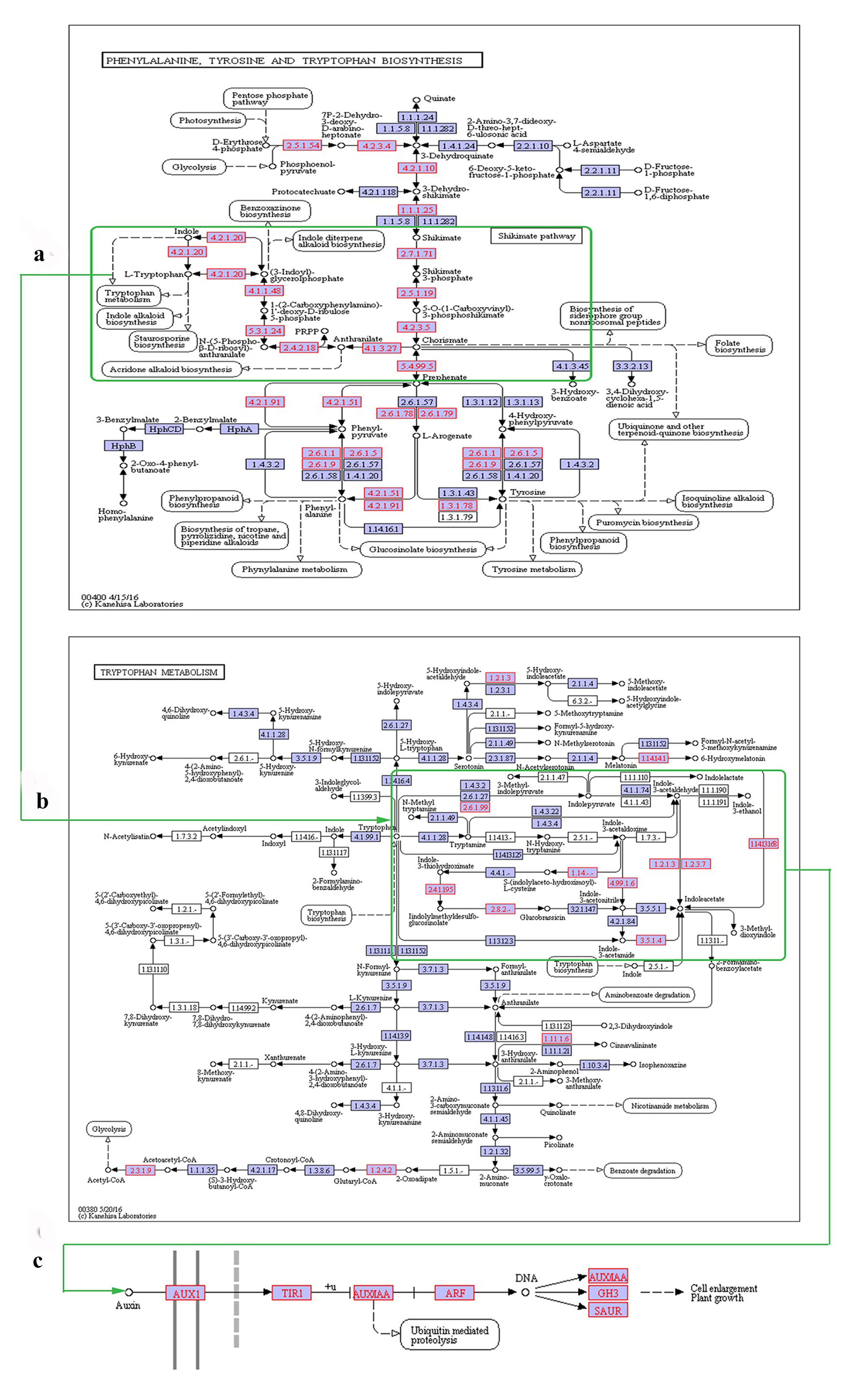

Supplement: Supplementary file 9 — Genes involved in the shikimate biosynthesis a), auxin biosynthesis b), and auxin signal transduction pathways c) within pistachio transcriptome, as indicated by pink color. (JPEG 1494 kb) [file 12864_2017_3989_MOESM9_ESM.jpg]

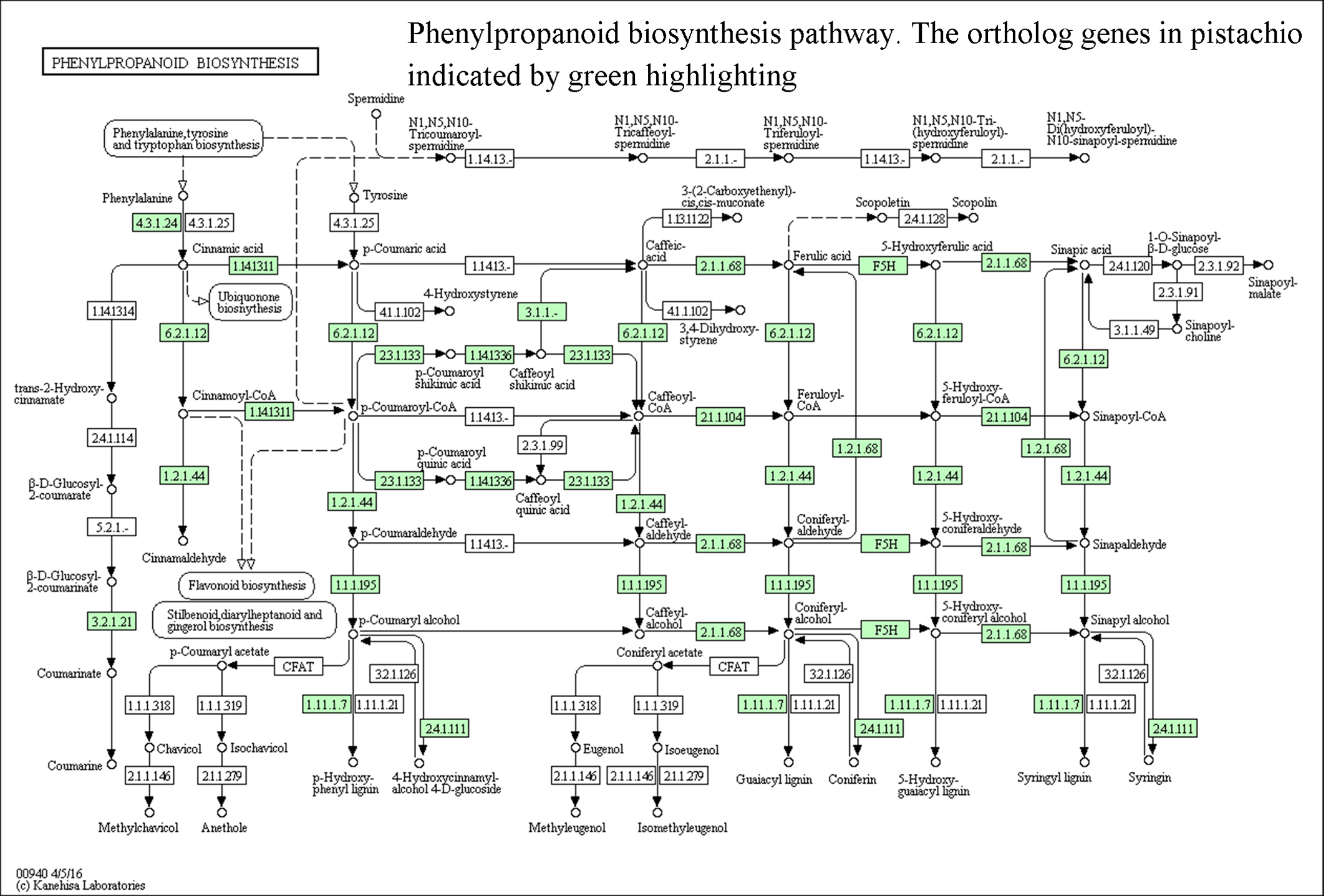

Supplement: Supplementary file 10 — Phenylpropanoid biosynthesis pathway. The ortholog genes in pistachio transcriptome indicated by green highlighting. (JPEG 704 kb) [file 12864_2017_3989_MOESM10_ESM.jpg]

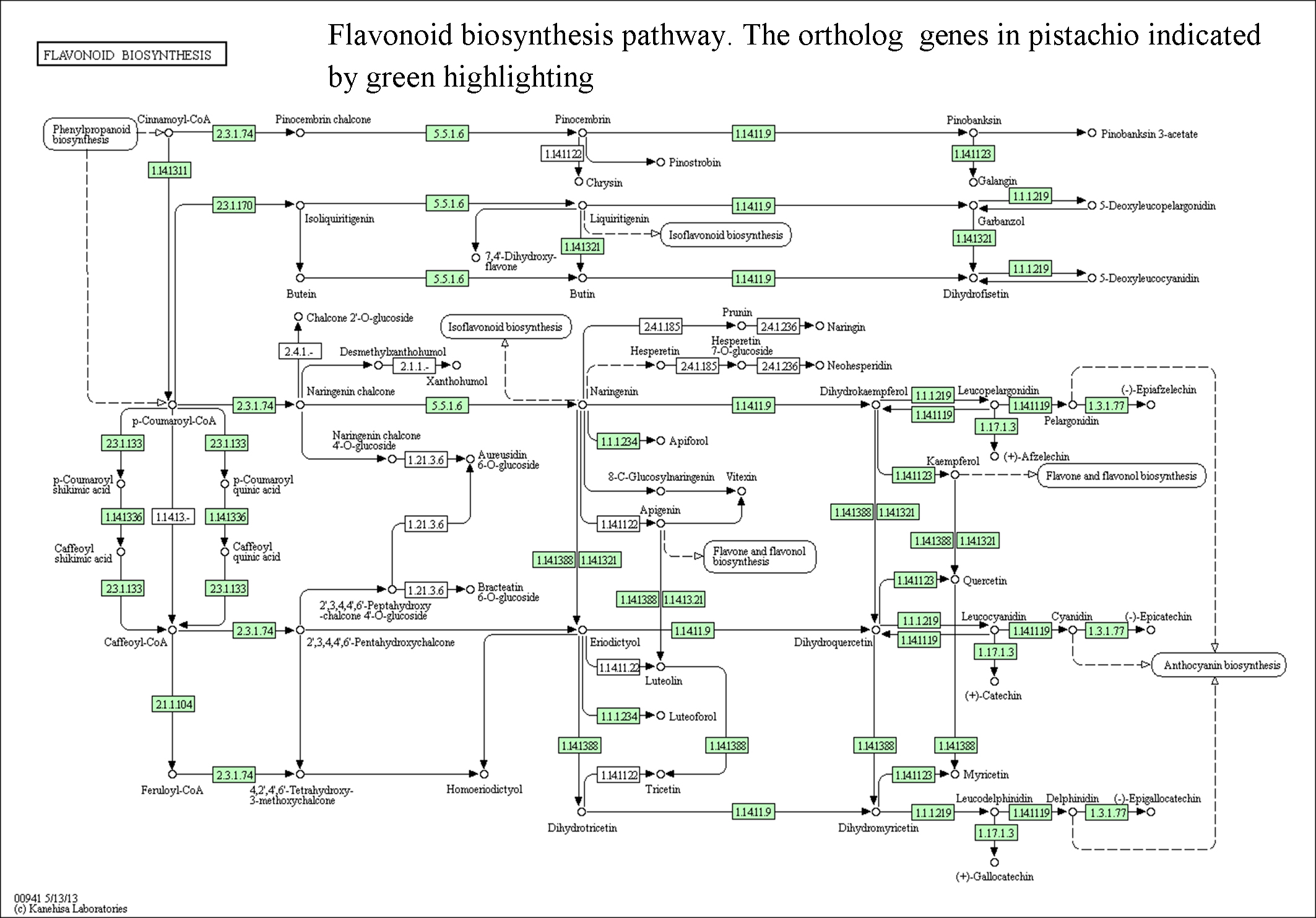

Supplement: Supplementary file 11 — Flavonoid biosynthesis pathway. The ortholog genes in pistachio transcriptome specified by green highlighting. (JPEG 608 kb) [file 12864_2017_3989_MOESM11_ESM.jpg]
